# *Talaromyces marneffei simA* Encodes a Fungal Cytochrome P450 Essential for Survival in Macrophages

**DOI:** 10.1128/mSphere.00056-18

**Published:** 2018-03-21

**Authors:** Kylie J. Boyce, David P. De Souza, Saravanan Dayalan, Shivani Pasricha, Dedreia Tull, Malcolm J. McConville, Alex Andrianopoulos

**Affiliations:** a School of Biosciences, The University of Melbourne, Melbourne, Australia; b Metabolomics Australia, Bio21 Institute, The University of Melbourne, Melbourne, Australia; c Department of Biochemistry and Molecular Biology, Bio21 Institute, The University of Melbourne, Melbourne, Australia; Carnegie Mellon University

**Keywords:** mycology, dimorphism, host-pathogen interactions

## Abstract

Fungi are adept at occupying specific environmental niches and often exploit numerous secondary metabolites generated by the cytochrome P450 (CYP) monoxygenases. This report describes the characterization of a yeast-specific CYP encoded by *simA* ("survival in macrophages"). Deletion of *simA* does not affect yeast growth at 37°C *in vitro* but is essential for yeast cell production during macrophage infection. The Δ*simA* strain exhibits reduced conidial germination and intracellular growth of yeast in macrophages, suggesting that the enzymatic product of SimA is required for normal fungal growth *in vivo*. Intracellular Δ*simA* yeast cells exhibit cell wall defects, and metabolomic and chemical sensitivity data suggest that SimA may promote chitin synthesis or deposition *in vitro*. *In vivo*, Δ*simA* yeast cells subsequently lyse and are degraded, suggesting that SimA may increase resistance to and/or suppress host cell biocidal effectors. The results suggest that *simA* synthesizes a secondary metabolite that allows *T. marneffei* to occupy the specific intracellular environmental niche within the macrophage.

**IMPORTANCE** This study in a dimorphic fungal pathogen uncovered a role for a yeast-specific cytochrome P450 (CYP)-encoding gene in the ability of *T. marneffei* to grow as yeast cells within the host macrophages. This report will inspire further research into the role of CYPs and secondary metabolite synthesis during fungal pathogenic growth.

## Abstract (precis)

This study in a dimorphic fungal pathogen uncovered a role for a yeast-specific cytochrome P450 (CYP)-encoding gene in the ability of *T. marneffei* to grow as yeast cells within the host macrophages. This report will inspire further research into the role of CYPs and secondary metabolite synthesis during fungal pathogenic growth.

## INTRODUCTION

Fungi are capable of producing a vast array of secondary metabolites, i.e., products of metabolism that are not essential for survival but that facilitate adaptation to specific environmental niches (reviewed in reference 1). Many secondary metabolites are modified by cytochrome P450s (CYPs), enzymes which belong to the superfamily of hemeproteins that commonly catalyze monooxygenase reactions. Although CYP genes can account for over 1% of a fungal genome, very few specific functions for CYPs have been uncovered. CYP genes are often found in clusters in the genome together with additional genes required for secondary metabolite synthesis such as polyketide synthase (PKS) and nonribosomal peptide synthase (NRPS) genes (2, 3). Interestingly, a number of CYPs have been identified that allow fungi to survive within the intracellular environment of a host during infection (4–6). After inhalation, fungal spores (conidia) are phagocytosed by alveolar macrophages and internalized to the mature phagolysosome where they are exposed to damaging reactive oxygen species (ROS) and reactive nitrogen species (RNS), low pH, and an array of hydrolytic enzymes. *NOR1*, a CYP-encoding gene in the fungal pathogen *Histoplasma capsulatum*, has been shown to detoxify the RNS nitric oxide (NO) produced by macrophages during infection (4, 5). On the other hand, CYP-encoding genes *ppoA*, *ppoB*, and *ppoC* in the monomorphic fungal pathogen *Aspergillus fumigatus* regulate the synthesis of oxylipins from polyunsaturated fatty acids, which have been shown to regulate the balance between asexual and sexual development and fatty acid regulation and to influence microbe-host interactions during infection (6–9).

Recently, a CYP exhibiting phase-specific expression in pathogenic yeast cells was identified in a microarray analysis in the dimorphic fungal pathogen *Talaromyces marneffei* (formerly named *Penicillium marneffei*). *T. marneffei* is a human-pathogenic fungus endemic to Southeast Asia that causes a fatal systemic mycosis. Like a number of other fungal pathogens, *T. marneffei* exhibits temperature-dependent dimorphic growth, alternating between saprophytic multicellular hyphae at 25°C and unicellular yeast at 37°C (10). *T. marneffei* infection is initiated by the inhalation of infectious propagules (conidia) produced by the hyphal growth form at 25°C, which are engulfed by alveolar macrophages in the host lung. Internalized conidia differentiate to yeast cells and proliferate within pulmonary alveolar macrophages of infected individuals (11). *T. marneffei* genes which are differentially regulated during saprophytic hyphal growth at 25°C, asexual development (conidiation) at 25°C, and *in vitro* yeast growth at 37°C have been identified using a genomic microarray (12). One of the 37°C yeast-specific microarray probes lies within the coding region of a gene encoding a CYP, *simA* ("survival in macrophages A"). Here, we investigated whether SimA is a potential virulence factor. In an initial analysis of the *T. marneffei* CYPome, we identified 116 CYPs representing over 1% of the total genome. A total of 18 CYP clusters were identified, and at least 8 of these are likely to be involved in secondary metabolite production. A total of 36 CYPs are predicted to be involved in the synthesis of secondary metabolites based on homology and/or proximity to genes of known function or to a PKS or NRPS gene. Interestingly, *simA* was not present in any of these CYP clusters, so a *simA* deletion strain was generated to further study its function. Deletion of *simA* does not affect *in vitro* yeast growth at 37°C but is essential for yeast cell production during *in vivo* macrophage infection. Loss of SimA results in loss of cell wall integrity during infection as well as in reduced survival *in vivo*. These results indicate that *simA* plays an important role during *T. marneffei* pathogenesis and that SimA may be involved in the production of a secondary metabolite that allows *T. marneffei* to occupy the intracellular vacuoles in host macrophages.

## RESULTS

### The identification of cytochrome P450s (CYPs) encoded within the *T. marneffei* genome.

All of the putative cytochrome P450s (CYPs) encoded within the *T. marneffei* genome were identified based on the presence of a putative P450 domain (PF00067) or by their similarity to orthologues of characterized CYPs, their assignment as orthologues of *Aspergillus nidulans* CYPs (3), and/or their association with CYP gene clusters. The identified CYPs were compared to those listed on the fungal cytochrome P450 database (http://p450.riceblast.snu.ac.kr/index.php?a=view) (13). A total of 116 CYP-encoding genes were identified in the *T. marneffei* genome (see Table S1 in the supplemental material). Although this number was identical to that seen in the fungal cytochrome P450 database, two CYPs in this database (EEA27060.1 and EEA23504.1) did not contain a putative P450 domain and were discounted, and there were two occurrences of P450s mapping to the same gene (EEA26095.1 and EEA26096.1; EEA25240.1 and EEA25395.1). Four CYPs not present in the fungal cytochrome P450 database were also identified (EEA18658.1, EEA18657.1, EEA18656.1, and EEA21701.1). The 116 CYPs in *T. marneffei* represent 69 CYP families and span over 1% of the total genome.

10.1128/mSphere.00056-18.3 TABLE S1  *T. marneffei* cytochrome P450s. Download TABLE S1, DOC file, 0.2 MB. Copyright © 2018 Boyce et al. 2018 Boyce et al. This content is distributed under the terms of the Creative Commons Attribution 4.0 International license.

The *T. marneffei* genome contains orthologues to seven CYPs that have been characterized in other fungal species (Table S2). These include five genes characterized in *A. nidulans*: *phacA* and *phacB*, which are required for phenylacetate utilization; *ppoC*, which is required for oxylipin biosynthesis; *ahbB*, which is involved in hyphal branching; and *bzuA*, which is required for benzamide utilization (7, 14–18). The *T. marneffei* genome also possessed orthologues to *Saccharomyces cerevisiae ERG5* and *ERG11*, which encode a C-22 sterol desaturase and lanosterol 14-alpha-demethylase, required for ergosterol biosynthesis (12, 19–21).

10.1128/mSphere.00056-18.4 TABLE S2  *T. marneffei* cytochrome P450s with characterized orthologues in other species. Download TABLE S2, DOC file, 0.04 MB. Copyright © 2018 Boyce et al. 2018 Boyce et al. This content is distributed under the terms of the Creative Commons Attribution 4.0 International license.

The *T. marneffei* genome did not possess orthologues of the other characterized *A. nidulans* CYPs, i.e., *ppoA* and *ppoB* (oxylipin biosynthesis); *apdB* and *apdE* (aspyridone biosynthesis); and *stcB*, *stcF*, *stcL* and *stcS* (sterigmatocystin synthesis) (7, 22–24). Interestingly, unlike *A. nidulans*, *T. marneffei* also lacks an orthologue to the third CYP in *S. cerevisiae*, Dit2p. *S. cerevisiae* sporulation-specific genes *DIT1* and *DIT2* catalyze a two-step reaction to produce a soluble ll-dityosine-containing precursor required for the production of the ll- and dl-dityrosine layer of the sexual spore wall (25, 26). *DIT1* and *DIT2* are clustered in *S. cerevisiae*, and *A. nidulans* contains both a *DIT1* orthologue (*ditA*; ANID_02705) and a *DIT2* orthologue (CYP56B1; ANID_02706), which are also clustered. The lack of these genes does not reflect an evolutionary consequence of an apparent lack of sexual reproduction in *T. marneffei*, as *T. stipitatus*, a close sexual relative of *T. marneffei*, also lacks both *DIT1* and *DIT2* orthologues. The *T. marneffei* genome also lacks a homologue of *NOR1*, a gene encoding a cytochrome P450 nitric oxide reductase in *Histoplasma capsulatum* (4, 5).

Putative functions can be postulated for some of the CYPs based on homology or close proximity to biosynthetic genes; PMAA_038590 encodes a CYP likely to be involved in quinic acid utilization, PMAA_065360 one likely to be involved in terpenoid biosynthesis, and PMAA_054540 and PMAA_071760 ones likely to be involved in siderophore biosynthesis (Table S3).

10.1128/mSphere.00056-18.5 TABLE S3  *T. marneffei* cytochrome P450s within gene clusters and those with predicted functions. Download TABLE S3, DOC file, 0.1 MB. Copyright © 2018 Boyce et al. 2018 Boyce et al. This content is distributed under the terms of the Creative Commons Attribution 4.0 International license.

### Identifying cytochrome P450 clusters in *T. marneffei.*

A CYP cluster has been previously defined as representing four or more cytochrome P450-encoding genes present within 100 kb of genome sequence or as groups that have fewer than seven genes between them (2, 3). Using these definitions, 18 CYP clusters were identified in *T. marneffei* (Table S3). Five of these CYP clusters contain genes of which no orthologue is present in *A. nidulans*. A total of 13 CYP gene clusters have been identified in *A. nidulans*, but the function of only 2 of these clusters, corresponding to sterigmatocystin biosynthesis and aspyridone biosynthesis, is known (3, 22, 27). Postulated functions for other clusters include ergot alkaloid biosynthesis and terpene synthesis (3). The *T. marneffei* genome lacks orthologues to the *A. nidulans* CYP cluster genes involved in aspyridone biosynthesis that are postulated to play a role in ergot alkaloid biosynthesis. The close proximity to specific metabolic genes allows prediction of the function for two *T. marneffei* CYP clusters: those related to ubiquinone biosynthesis and gliotoxin production (Table S3) (28). Many of the gene clusters are likely to play a role in the production of as-yet-undefined secondary metabolites. For example, one five-CYP cluster is in close proximity to the orthologue of toxin biosynthesis protein Tri7 (PMAA_043560), required for trichothecene mycotoxin biosynthesis in *Fusarium graminearum*, and bZIP transcription factor CpcA, required for sirodesmin production in *Leptosphaeria maculans* (29, 30).

### The identification of CYPs involved in secondary metabolite synthesis.

To identify additional CYPs potentially required for secondary metabolite synthesis, the proximity of polyketide synthase (PKS) and nonribosomal peptide synthase (NRPS) genes was analyzed. Eighteen CYPs were in close proximity to a PKS or NRPS gene, suggesting a role in secondary metabolite synthesis. Twelve of these were in five CYP clusters, suggesting that these clusters have a putative role in secondary metabolite synthesis (Table S3). In addition, the secondary metabolite unique regions finder (SMURF) (http://jcvi.org/smurf/index.php) was also used to identify CYPs involved in secondary metabolite synthesis. Nine secondary metabolite clusters predicted by Smurf contained a CYP (Table S3). Eight of these were identified in the prior analysis using proximity to a PKS and NRPS. Therefore, a total of 31 CYPs are predicted to be involved in the synthesis of secondary metabolites based on homology and/or proximity to genes of known function or to a PKS or NRPS gene. At least seven CYP clusters (20 CYPs) in *T. marneffei* are likely to be involved in secondary metabolite production.

### The identification of a cytochrome P450, encoded by *simA*, specifically expressed during yeast growth at 37°C.

We have previously shown that *simA* is selectively expressed during *in vitro* yeast growth (12). The 538-amino-acid predicted protein contains a cytochrome P450 domain at amino acids 50 to 507 (http://pfam.xfam.org/family/PF00067). This domain contains a predicted oxygen binding and activation motif at amino acids 321 to 326 (AGXXTT) and the conserved EXXR motif (amino acids 379 to 382), the PER(W) domain (amino acids 435 to 438), and the FXXGXXXCXG heme binding domain (amino acids 470 to 479) characteristic of CYPs (3). SimA was shown to be a member of the CYP548 family based on the best hit of Nelson’s classification (family members share >40% amino acid identity). SimA showed no homology to CYPs characterized in other organisms, and a putative function could not be postulated based on close proximity to characterized genes. SimA is not present in a CYP cluster or in close proximity to a PKS or NRPS gene, suggesting that it may not be involved in the production of a secondary metabolite.

**FIG 1:**
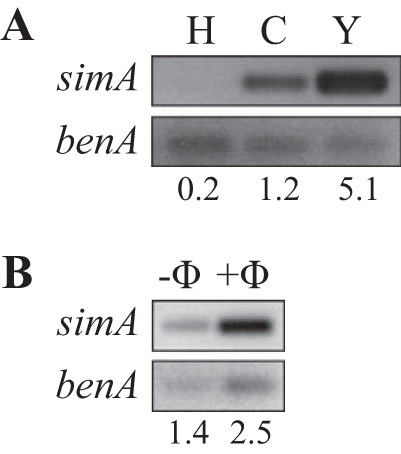
Expression of *simA*. (A) Reverse transcriptase PCR (RT-PCR) of *simA* and a *benA* loading control performed on RNA isolated from wild-type vegetative hyphae grown for 2 days in liquid medium at 25°C, asexual development (conidiation) cultures grown for 7 days on solid medium at 25°C, and yeast cells grown for 6 days in liquid medium at 37°C. A *simA* transcript could not be detected during vegetative hyphal growth at 25°C. *simA* is expressed during asexual development at 25°C and highest during yeast growth at 37°C. Relative intensity values, adjusted using the loading controls, are indicated below the lanes. (B) RNA was isolated from wild-type *T. marneffei* incubated in macrophage medium alone for 24 h at 37°C (-Φ) or from infected LPS-activated J774 murine macrophages 24 h postinfection at 37°C (+Φ). The amount of *simA* transcript was increased in cells isolated from infected macrophages. Relative intensity values, adjusted using the loading controls, are indicated below the lanes.

To confirm the yeast-specific expression of *simA*, RNA was isolated from wild-type vegetative hyphae grown for 2 days in liquid medium at 25°C, asexual development (conidiation) cultures grown for 7 days on solid medium at 25°C, and yeast cells grown for 6 days in liquid medium at 37°C. Reverse transcriptase PCR (RT-PCR) showed that *simA* was expressed during asexual development at 25°C and during yeast growth at 37°C (Fig. 1A). A *simA* transcript could not be detected during vegetative hyphal growth at 25°C (Fig. 1A). The level of expression was highest during yeast growth, consistent with the original microarray data.

*T. marneffei* infection is hypothesized to occur by inhalation of conidia, which are phagocytosed by pulmonary alveolar macrophages. Once phagocytosed, conidia germinate into pathogenic yeast cells that proliferate within the macrophage (10). To examine if *simA* expression is induced during infection, RNA was isolated from wild-type *T. marneffei* incubated for 24 h at 37°C in macrophage media alone or from infected lipopolysaccharide (LPS)-activated J774 murine macrophages incubated for 24 h postinfection at 37°C (Materials and Methods). The amount of *simA* transcript was increased in cells isolated from infected macrophages, suggesting that *simA* expression is induced during infection (Fig. 1B).

### SimA is localized to the endoplasmic reticulum.

**FIG 2:**
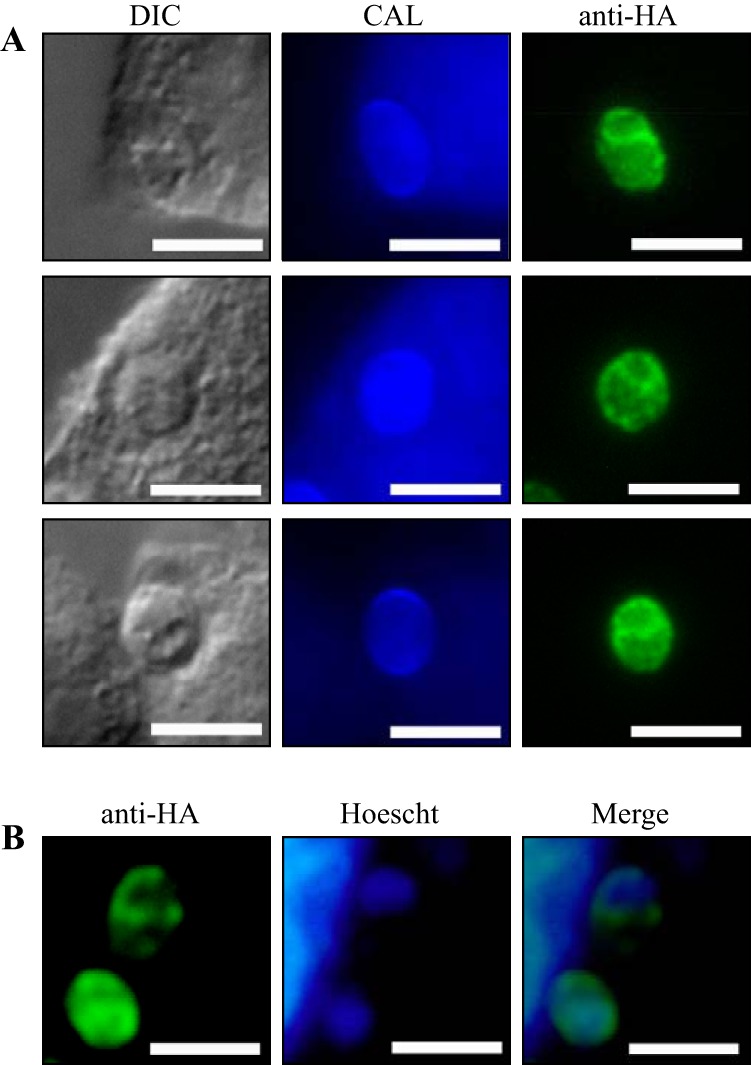
SimA localization in yeast cells during macrophage infection. Anti-HA immunostaining was performed on macrophages infected with *simA*^+^ HA conidia 24 h postinfection. (A) Calcofluor (CAL) staining of *T. marneffei* yeast cells during infection. Anti-HA immunostaining shows SimA localized in the cytoplasm. (B) Costaining with Hoechst 33258 (Hoescht) shows that the localization is perinuclear, consistent with localization in the ER. Images were captured using differential interference contrast (DIC) or with epifluorescence to observe calcofluor-stained fungal cell walls (CAL) or Hoechst 33258-stained nuclei (Hoescht). Scale bars, 10 µm.

Eukaryotic CYPs are typically membrane bound and anchored on the cytoplasmic surface of the endoplasmic reticulum (ER) through a short N-terminal hydrophobic sequence (3, 31). Using Target P (http://www.cbs.dtu.dk/services/TargetP/), SimA is predicted to be ER localized (32). The predicted SimA protein sequence contains a 30-amino-acid hydrophobic N-terminal ER signal sequence but no C-terminal ER retention signal. To investigate the localization of SimA, a triple-hemagglutinin (HA) tag was inserted between amino acids 527 and 528, which is a nonconserved region positioned after the cytochrome P450 domain in the C terminus. *T. marneffei* strain G147 (*niaD pyrG*) was transformed with the *simA*::*HA* construct, and integration was confirmed by Southern blot analysis (Materials and Methods). Anti-HA immunostaining was performed on macrophages infected with *simA*::*HA* conidia 24 h postinfection (Materials and Methods). The tagged SimA showed overlapping perinuclear staining with Hoescht 33258, consistent with localization in the ER (Fig. 2).

### Deletion of *simA* did not affect hyphal growth or asexual development at 25°C.

To investigate the role of *simA* in *T. marneffei* growth and development, the *simA* gene was deleted. A split marker *simA* deletion construct, which deleted nucleotides −37 to +1760 of *simA*, was used to transform *T. marneffei* strain G526 (Δ*pkuA niaD pyrG areA*^*−*^), and *pyrG*-positive (*pyrG*^*+*^) transformants were selected (Materials and Methods). These transformants were screened by genomic Southern blotting, and one Δ*simA* transformant was identified (G559) which possessed a restriction pattern consistent with replacement of *simA* by a single copy of *pyrG* at the genomic locus (data not shown). To complement the Δ*simA* mutation, the Δ*simA* transformant was transformed with a *simA barA*^*+*^ plasmid, generating a Δ*simA simA*^*+*^ (strain G893) transformant (Materials and Methods). As we subsequently found that the Δ*pkuA* genetic background is associated with defects in genome stability (33), *simA* was also deleted in strain G816 (Δ*ligD niaD pyrG*). The phenotypes of Δ*simA* strains in this background were compared to those of the original deletion strain and found to be phenotypically indistinguishable.

At 25°C, wild-type *T. marneffei* grows as highly polarized vegetative hyphae which differentiate asexual structures (conidiophores). Colonies of the Δ*simA* and Δ*simA simA*^*+*^ strains were indistinguishable from the wild type after 10 days of growth at 25°C. To investigate if hyphae and conidiophores produced by the Δ*simA* mutant possessed wild-type morphology, the wild-type, Δ*simA*, and Δ*simA simA*^*+*^ strains were grown on agar-coated slides (1% and 0.1% glucose) for 4 days at 25°C and stained with calcofluor (CAL) to visualize cell walls and with Hoescht 33258 to observe nuclei. Hyphae and conidiophores of the Δ*simA* and Δ*simA simA*^*+*^ strains were indistinguishable from those seen with the wild-type strain, indicating that SimA is not required for hyphal growth and development (see Fig. S1 in the supplemental material).

10.1128/mSphere.00056-18.1 FIG S1  The Δ*simA* mutant produces hyphae and conidiophores with wild-type morphology at 25°C. Wild-type (*simA*^*+*^) and Δ*simA* strains were grown on ANM containing 1% or 0.1% glucose for 4 days at 25°C. Images were captured using differential interference contrast (DIC) or epifluorescence to observe calcofluor-stained fungal cell walls (CAL) and Hoechst 33258-stained nuclei (Hoescht). Scale bars, 20 µm. Download FIG S1, PDF file, 0.8 MB. Copyright © 2018 Boyce et al. 2018 Boyce et al. This content is distributed under the terms of the Creative Commons Attribution 4.0 International license.

### Deletion of *simA* did not affect *in vitro* yeast growth at 37°C but was essential for yeast cell production during macrophage infection.

**FIG 3:**
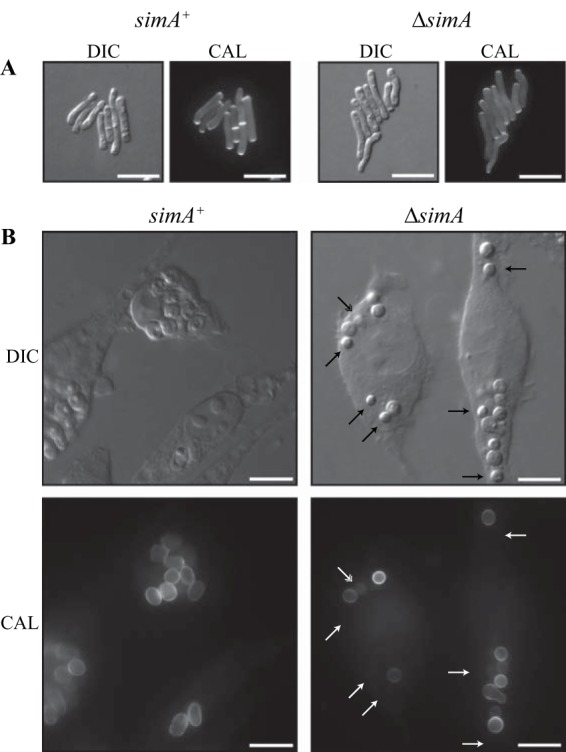
SimA is required for yeast growth *in vivo* but not *in vitro*. (A) *simA*^+^ and Δ*simA* strains grown *in vitro* on BHI medium for 5 days at 37°C. Numerous Δ*simA* yeast cells were observed after 5 days, and the cells were morphologically indistinguishable from the wild-type (*simA*^*+*^) cells. (B) Macrophages infected with *simA*^+^ and Δ*simA* conidia 24 h postinfection. After 24 h, numerous yeast cells dividing by fission were observed in macrophages infected with wild-type (*simA*^*+*^) conidia. In contrast, Δ*simA* conidia remained mostly ungerminated after 24 h. A proportion of those conidia did not stain with calcofluor (indicated by arrows) and appeared to have lost cellular integrity (double-headed arrow). Images were captured using differential interference contrast (DIC) or with epifluorescence to observe calcofluor-stained fungal cell walls (CAL). Scale bars, 10 µm (A) and 20 µm (B).

After 6 days of growth at 37°C on brain heart infusion (BHI) medium, wild-type *T. marneffei* produces brown (melanized), surface-convoluted, yeast-like colonies. Compared to the wild-type and Δ*simA simA*^*+*^ strains, the Δ*simA* strain showed reduced pigmentation after 6 days at 37°C on BHI medium (Fig. S2). To assess whether the reduced pigmentation was due to an inability to produce pyomelanin or l-3,4-dihydroxyphenylalanine (L-DOPA) melanin, strains were also grown at 37°C on medium with tyrosine as the sole nitrogen source and on DOPA medium (34). Although colonies of the Δ*simA* strain possessed differences in colony morphology on these media, melanization was not affected, suggesting that the reduced pigmentation observed on BHI medium was not a result of an inability to produce pyo- or DOPA-melanin (Fig. S2). To observe yeast cell morphogenesis *in vitro*, the wild-type, Δ*simA*, and Δ*simA simA*^*+*^ strains were inoculated onto agar-coated slides and incubated for 6 days at 37°C. Wild-type conidia germinate at 37°C to produce polarized arthroconidiating hyphae, in which nuclear division and septation become coupled and double septa are laid down, and fragmentation occurs along this plane to liberate uninucleate yeast cells, which consequently divide by fission. After 6 days at 37°C, arthroconidiating hyphae and numerous yeast cells were observed for the wild-type, Δ*simA*, and Δ*simA simA*^*+*^ strains and all strains were the same with respect to morphology and nuclear index value (Fig. 3A).

10.1128/mSphere.00056-18.2 FIG S2  The Δ*simA* mutant does not display a reduction in pyomelanin or DOPA melanization at 37°C. Growth of the wild-type, Δ*simA*, and Δ*simA simA*^+^ strains at 37°C on BHI medium after 5 days (A), on tyrosine as the sole nitrogen source after 14 days (B), and on L-DOPA medium after 14 days (C). Download FIG S2, PDF file, 0.4 MB. Copyright © 2018 Boyce et al. 2018 Boyce et al. This content is distributed under the terms of the Creative Commons Attribution 4.0 International license.

To investigate if *simA* is required for the production of yeast cells *in vivo*, LPS-activated J774 murine macrophages grown at 37°C were infected with wild-type, Δ*simA*, and Δ*simA simA*^*+*^ conidia and infections were monitored microscopically at 2, 24, and 48 h postinfection. Calcofluor staining was performed to allow visualization of fungal cell walls. After 2 h, macrophages coincubated with wild-type conidia contained numerous phagocytosed conidia (216 ± 15.7 conidia per 100 macrophages). Δ*simA* and Δ*simA simA*^*+*^ conidia were phagocytosed by macrophages at the same level as wild-type conidia (212 ± 7.33 conidia per 100 macrophages for the Δ*simA* strain and 214 ± 6.58 conidia per 100 macrophages for the Δ*simA simA*^*+*^ strain). At 24 h postinfection, some (5.90% ± 1.36%) wild-type conidia remained ungerminated. However, the majority (94.1% ± 1.36%) had germinated directly into yeast cells by isotropic expansion (Fig. 3B). A small proportion (0.60% ± 0.60%) of conidia germinated by polarized extension to form a germ tube, and these germlings subsequently broke apart to produce yeast cells. In contrast, phagocytosed Δ*simA* conidia remained predominately (63.9.0% ±5.50%) ungerminated. Some (1.45% ± 1.17%) germlings and reduced (34.7% ± 5.10%) numbers of yeast cells were observed. Reintroduction of *simA* restored the wild-type germination phenotype (ungerminated conidia, 9.33% ± 1.35%; germlings, 6.10% ± 0.80%; yeast cells, 84.6% ± 1.88%). The decrease in Δ*simA* conidial germination was specific to macrophage infection, as the conidia that had germinated for 15 h *in vitro* at 37°C showed germination kinetics indistinguishable from wild-type-strain kinetics (75.6% ± 4.90% germinated for the *simA*^*+*^ strain, and 70.7% ± 6.69% germinated for the Δ*simA* strain).

**FIG 4:**
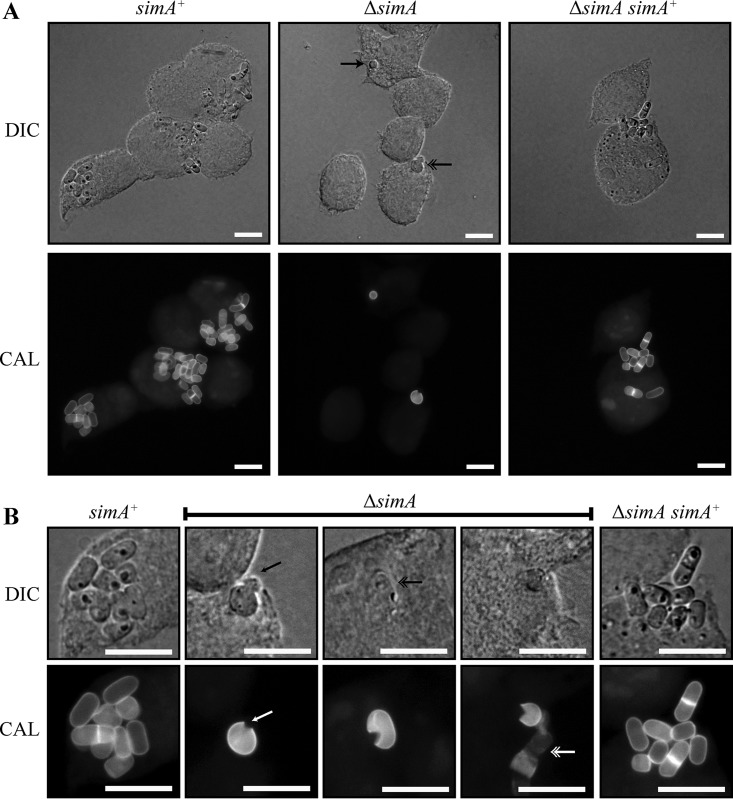
The Δ*simA* strain shows less cellular proliferation during macrophage infection and ruptured yeast cells. (A) Macrophages infected with *simA*^+^, Δ*simA*, and Δ*simA simA*^+^ conidia 48 h postinfection. After 48 h, numerous yeast cells dividing by fission were observed in macrophages infected with wild-type (*simA*^*+*^) or Δ*simA simA*^+^ conidia. The Δ*simA* strain showed less cellular proliferation within macrophages than the wild-type (*simA*^*+*^) and Δ*simA simA*^+^ strains. Some Δ*simA* conidia remained ungerminated (black arrowhead), but a number had germinated into yeast cells (black double arrowhead). Yeast cells appeared ruptured and to be releasing cellular contents (black double arrowhead). (B) Unlike wild-type (*simA*^*+*^) and Δ*simA simA*^+^ yeast cells, Δ*simA* yeast cells were often ruptured (white arrowhead) and leaking their cellular contents (black arrowhead). Cells were not clearly visible under DIC conditions (black double arrowhead), and degraded fungal cellular material was observed (white double arrowhead). Images were captured using differential interference contrast (DIC) or with epifluorescence to observe calcofluor-stained fungal cell walls (CAL). Scale bars, 10 µm.

Wild-type fungi grew as yeast cells 48 h postinfection, dividing by fission (ungerminated conidia, 0.33% ± 0.33%; germlings, 1.03% ± 1.03%; yeast cells, 98.6% ± 0.91%) (Fig. 4A). At that time point, a proportion of the Δ*simA* conidia remained ungerminated (14.6% ± 4.03%) or were visible as germlings (16.1% ± 3.86%). Reintroduction of *simA* effectively complemented this phenotype (ungerminated conidia, 0.60% ± 0.60%; germlings, 3.23% ± 3.23%; yeast cells, 96.2% ± 3.83%) (Fig. 4A). While the Δ*simA* strain produced some yeast cells (69.3% ± 7.35%), the proliferation of those cells was greatly reduced compared to the wild-type strain and the complemented strain (Fig. 4A). Specifically, while majorities of macrophages infected with the wild-type strain and the Δ*simA simA*^*+*^ strain contained more than 2 yeast cells (67.6% ± 3.63% and 68.4% ± 1.96%, respectively), only 21.0% ± 1.21% of macrophages infected with the Δ*simA* strain had more than 2 yeast cells.

### SimA is required for cell wall integrity during infection.

Calcofluor straining of intracellular fungi indicated that the Δ*simA* conidia and germlings might have had a defect in cell wall synthesis (Fig. 3B). Specifically, while intracellular wild-type and Δ*simA simA*^*+*^ yeast cells were labeled with calcofluor (98.4% ± 1.05% and 99.3% ± 0.67%, respectively), only 59.1% ± 9.31% of intracellular Δ*simA* yeast cells stained with this cell wall dye. In addition, some non-calcofluor-staining conidia appeared degraded and devoid of cellular content. The loss of calcofluor staining was observed only during macrophage infection. Conidial suspensions of the wild-type, Δ*simA*, and Δ*simA simA*^*+*^ strains all stained strongly with calcofluor. Interestingly, when LPS-activated J774 murine macrophages were infected with wild-type, Δ*simA*, and Δ*simA simA*^*+*^ conidia and observed 2 h postinfection, the numbers of calcofluor-stained cells did not differ between the strains (96.4% ± 1.11% of the wild-type, 100% ± 0.00% of the Δ*simA simA*^*+*^, and 96.3% ± 1.33% of the Δ*simA* conidia stained with calcofluor), suggesting that the decreased calcofluor staining of the Δ*simA* cells had occurred upon prolonged intracellular incubation within macrophages. After 48 h, ungerminated Δ*simA* conidia that did not stain with calcofluor were no longer observed. It is likely that these cells had been degraded by the macrophage. The small numbers of yeast cells which were present after 48 h were often not clearly visible under differential interference contrast (DIC) microscopy and were ruptured and leaking their cellular contents (Fig. 4B). Degraded fungal cellular material staining faintly with calcofluor was also observed in macrophages (Fig. 4B).

**FIG 5:**
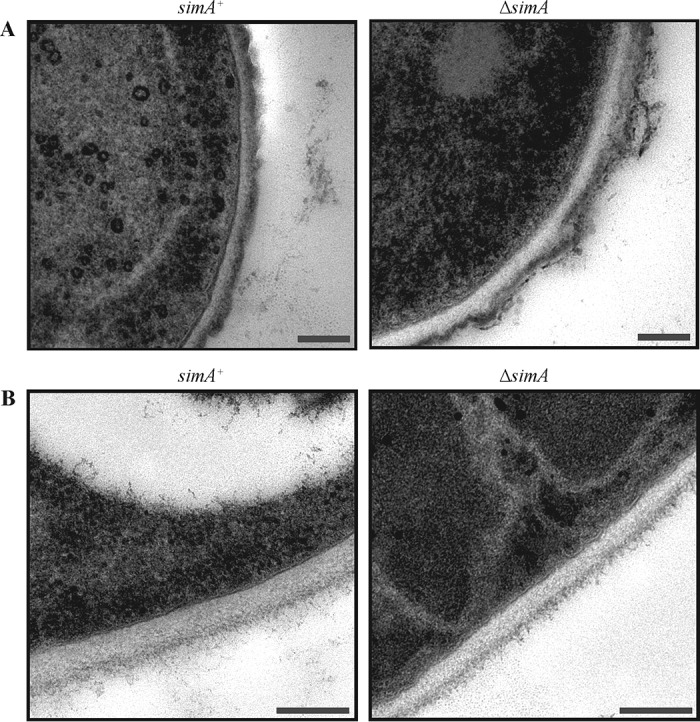
Conidia of the Δ*simA* mutant display cell wall defects. (A and B) Transmission electron microscopy of wild-type (*simA*^*+*^) and Δ*simA* conidia (ANM medium for 12 days at 25°C) (A) and yeast cells grown *in vitro* (BHI medium for 5 days at 37°C) (B). (A) The cell wall of wild-type (*simA*^*+*^) conidia appears as three layers: a thin dense layer which lies directly adjacent to the lipid bilayer, a thick nondense layer in the middle layer, and an outer electron-dense layer which is slightly uneven. Compared to the wild-type results, the middle and outer layers of Δ*simA* conidia are unevenly distributed. (B) The cell wall of wild-type (*simA*^*+*^) yeast cells grown *in vitro* appears as two distinct layers: a thick dense layer lying directly adjacent to the lipid bilayer and a denser, unevenly distributed outer layer. The cell wall of Δ*simA* yeast cells *in vitro* appears indistinguishable from the wild-type (*simA*^*+*^) cell wall. Scales bars, 100 nm (A) and 0.2 µm (B).

**FIG 6:**
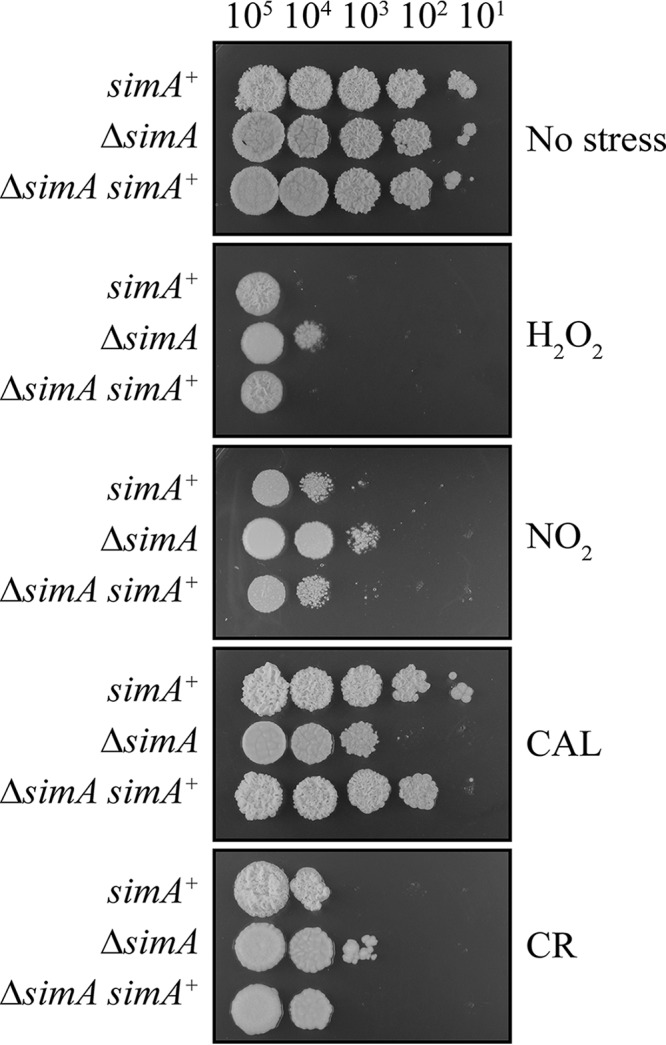
Δ*simA* is sensitive to cell wall-disrupting agents and resistant to oxidizing agents at 37°C. Serial dilutions of wild-type, Δ*simA*, and Δ*simA simA*^+^ conidial suspensions were dropped onto SD medium-(NH_4_)_2_SO_4_ plates containing 30 μg/μl calcofluor (CAL), 10 μg/μl Congo red (CR), 1 mM H_2_O_2_, or 0.5 mM NO_2_ and incubated for 5 days at 37°C.

To further define the nature of the cell wall defect in Δ*simA* conidia, sections of wild-type and Δ*simA* conidia and of *in vitro* yeast cells were analyzed by transmission electron microscopy (TEM). The cell wall of wild-type conidia appears as three layers: a thin dense layer which lies directly adjacent to the lipid bilayer of the cell membrane, a thick nondense layer in the middle, and an outer electron-dense layer which is slightly uneven (Fig. 5A). All the cell wall layers were visible in the Δ*simA* conidia; however, the middle and outer layers were unevenly distributed, indicating that the Δ*simA* conidia might have had defects in the conidial cell wall (Fig. 5A). This was further confirmed by plating conidia *in vitro* at 37°C on media containing increasing concentrations of calcofluor and Congo red, a commonly used indication of cell wall defects (Materials and Methods). The Δ*simA* mutant was more sensitive and resistant to calcofluor and Congo red at 37°C than the parental wild-type control and the Δ*simA simA*^*+*^ mutant, respectively (Fig. 6).

In contrast to the conidia, the cell wall of wild-type *in vitro* yeast cells appeared as only two layers: a thick electron-translucent layer which lies directly adjacent to the lipid bilayer and an outer electron-dense layer (Fig. 5B). No differences between the cell walls of wild-type and Δ*simA in vitro* yeast cells could be detected (Fig. 5B).

**FIG 7:**
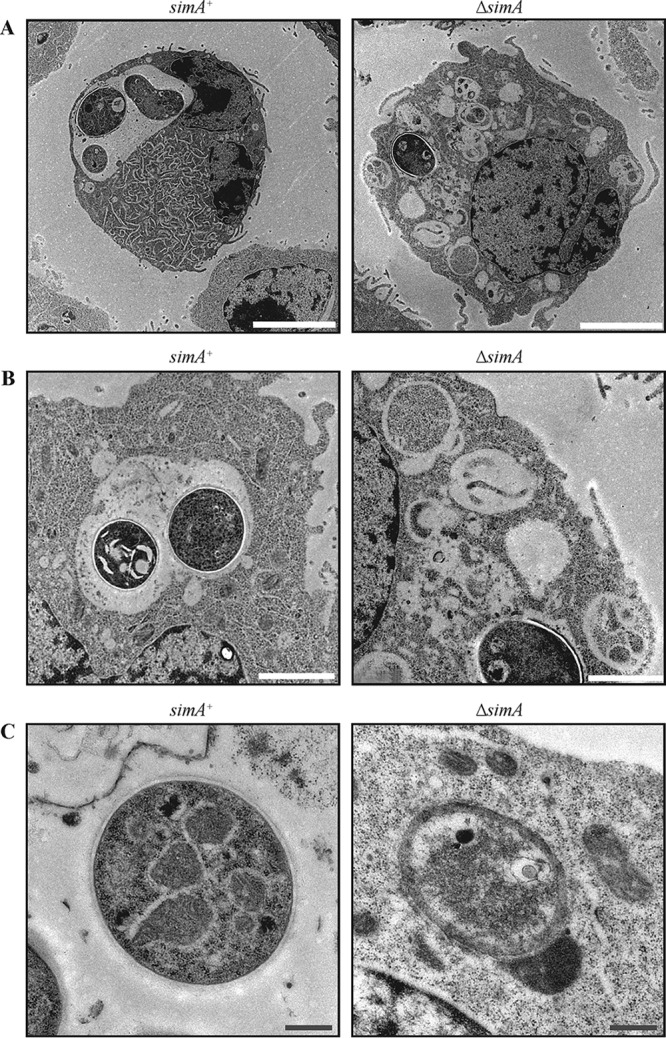
Δ*simA* yeast cells appear degraded *in vivo*. Transmission electron microscopy images of the wild-type (*simA*^*+*^) and Δ*simA* strains 24 h postinfection of macrophages are shown. (A) After 24 h postinfection of macrophages, wild-type (*simA*^*+*^) yeast cells were observed within a macrophage phagosome. In contrast, the ungerminated Δ*simA* conidia were not encompassed within an intracellular macrophage compartment. (B) In contrast to macrophages infected with the wild-type strain, macrophages infected with Δ*simA* conidia contained numerous degraded cells. (C) The cell wall of wild-type (*simA*^*+*^) yeast cells appeared as at least two distinct defined layers: a thick dense layer lying directly adjacent to the lipid bilayer and a denser, unevenly distributed outer layer. Some Δ*simA* conidia lacked clearly defined cell wall layers. Scale bars, 5 µm (A), 2 µm (B), and 0.5 µm (C).

To examine whether cell wall defects would be observable in the Δ*simA* strain during macrophage infection, LPS-activated J774 murine macrophages were infected with wild-type or Δ*simA* conidia and observed by transmission electron microscopy 24 h postinfection (Materials and Methods). Wild-type yeast cells were observed within a macrophage phagosome (Fig. 7A). The two cell wall layers were clearly visible, as were cellular organelles and septa in dividing cells (Fig. 7). In marked contrast, intracellular Δ*simA* conidia lacked well-defined cell wall layers and appeared devoid of cellular structures and organelles (Fig. 7). In addition, many degraded conidia were observed in which cell walls and cellular contents were absent (Fig. 7A and B).

### *simA* is not required to block phagolysosomal maturation.

**FIG 8:**
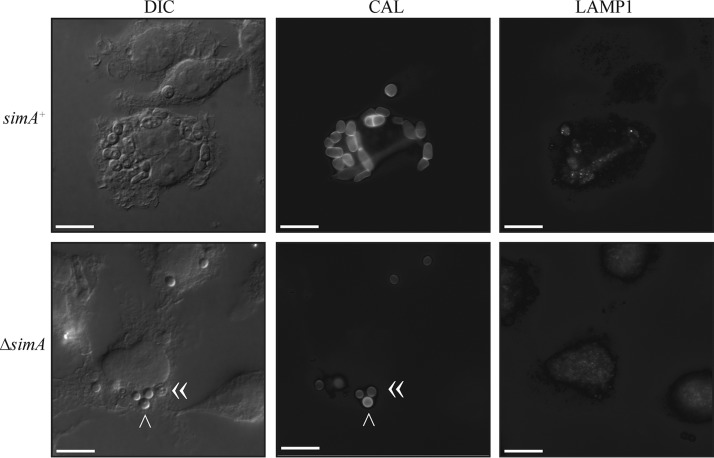
Deletion of *simA* does not result in increased phagolysosomal maturation. LPS-activated J774 murine macrophages infected with wild-type or Δ*simA* conidia 24 h postinfection were labeled with the lysosomal marker LAMP1. Wild-type or Δ*simA* conidia did not colocalize with LAMP1. The arrowhead and double arrowheads indicate a Δ*simA* conidium that stained with calcofluor white and Δ*simA* conidia that were not stained, respectively. Images were captured using differential interference contrast (DIC) or with epifluorescence to observe calcofluor-stained fungal cell walls (CAL) or LAMP1. Scale bars, 20 µm.

One possible explanation for the absence of calcofluor staining and the presence of degraded Δ*simA* cells *in vivo* is that SimA normally blocks phagolysosomal maturation, a survival strategy utilized by some bacterial and fungal pathogens in order to survive phagocytic destruction (reviewed in reference 35). To examine whether phagolysosomal formation was increased in the Δ*simA* mutant compared to the wild type, LPS-activated J774 murine macrophages were infected with wild-type or Δ*simA* conidia and labeled 24 h postinfection with the lysosomal markers LAMP1 and cathepsin D by immunohistochemistry. Interestingly, unlike the *Talaromyces stipitatus* positive control, neither the wild-type nor the Δ*simA* yeast cells were located in LAMP1-positive and cathepsin D-positive compartments after 24 h, suggesting that *T. marneffei* yeast cells severely delay or block late phagosome maturation and fusion to lysosomes (Fig. 8 and data not shown). Δ*simA* conidia and germlings were also not colocalized with LAMP1 or cathepsin D, suggesting that deletion of *simA* does not result in increased phagolysosomal maturation (Fig. 8, bottom row, and not shown). These observations suggest that the increased sensitivity of the Δ*simA* mutant to host microbicidal processes does not reflect differential transport to the mature lysosome.

### The Δ*simA* mutant is resistant to oxidative stress.

To investigate if the deletion of *simA* results in increased sensitivity to oxidative stress, the wild-type, Δ*simA*, and Δ*simA simA*^*+*^ strains were plated on media containing increasing concentrations of H_2_O_2_ and NO_2_ at 37°C. Unexpectedly, the Δ*simA* mutant showed increased resistance to both H_2_O_2_ and NO_2_ compared to the wild-type and Δ*simA simA*^*+*^ strains at 37°C (Fig. 6). To assess whether this was a more general phenomenon, the wild-type and Δ*simA* strains were also plated on media containing H_2_O_2_ and NO_2_ at 25°C and under salt stress (NaCl) and osmotic stress (sorbitol) conditions at both 25°C and 37°C. The Δ*simA* mutant was indistinguishable from the wild-type and Δ*simA simA*^*+*^ strains under those conditions (not shown).

### The Δ*simA* mutant possesses alterations in cellular metabolism.

**FIG 9:**
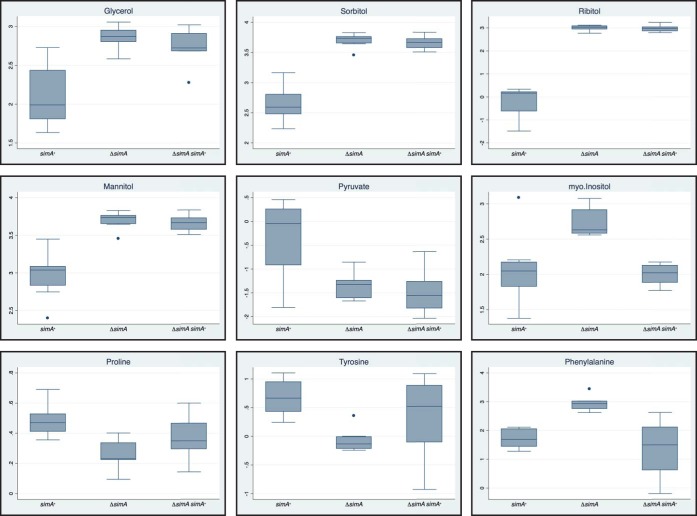
Box plots of a selection of polar metabolites exhibiting significant differences between the wild-type and Δ*simA* mutant strains. The dots represent data points which are considered outliers.

To further understand the function of SimA and possible substrates for this CYP, polar metabolomic extracts of mid-log-phase yeast cells of the wild-type, Δ*simA*, and Δ*simA simA*^*+*^ strains grown at 37°C were analyzed by gas chromatography-mass spectrometry (GC-MS) (Materials and Methods). The abundance of metabolites was determined for each strain, and metabolites were identified where possible by comparison to mass spectral libraries (Materials and Methods). Principal-component analysis (PCA) of the data revealed significant differences between the metabolomic profiles of the strains, indicating that the reintroduction of *simA*^*+*^ in the Δ*simA simA*^*+*^ complementation strain did not result in a complete recovery of the wild-type metabolite profile but rather in an profile intermediate between the wild-type and Δ*simA* profiles. This was readily observable by examination of individual metabolites such as proline (Fig. 9). The lack of full complementation was likely due to a position effect resulting from integration location of the complementation construct or from the presence of an incomplete promoter. Paired Student’s *t* tests were performed to identify those metabolites whose abundance significantly differed between strains (*P* value < 0.05) (Table S4).

10.1128/mSphere.00056-18.6 TABLE S4  Polar metabolites showing significant differences between the wild-type and Δ*simA* mutant strains in the levels of mid-log-phase yeast cells. Download TABLE S4, DOC file, 0.1 MB. Copyright © 2018 Boyce et al. 2018 Boyce et al. This content is distributed under the terms of the Creative Commons Attribution 4.0 International license.

Compared to the wild type, the Δ*simA* mutant exhibited a decrease in the levels of glycerol-3-phosphate with a concomitant increase in levels of glycerol that was suggestive of increased flux into glycerol production (Fig. 9). In fungi, glycerol is rapidly accumulated to cope with high external osmolarity (reviewed in reference 36). Elevated glycerol could reflect a response to loss of cell wall integrity and/or a need to maintain osmotic homeostasis (37). Increased flux into glycerol synthesis could also occur at the expense of glycolysis, as shown by reduced levels of pyruvate in the Δ*simA* mutant (Table S4) (Fig. 9). In addition to increased glycerol levels, the Δ*simA* mutant exhibited an increase in the levels of other sugar alcohols whose levels are known to be elevated under conditions of osmotic stress such as sorbitol, ribitol, and mannitol (38) (Table S4). The levels of 16 amino acids and of the alanine analogue aminoisobutyric acid were also either increased (threonine, glutamate, phenylalanine, aspartate, and aminoisobutyric acid) or decreased (alanine, valine, proline, glycine, histidine, serine, isoleucine, methionine, tryptophan, leucine, asparagine, and tyrosine) in the Δ*simA* mutant compared to the wild type (Fig. 9; see also Table S4). Changes in amino acid synthesis (or uptake) could reflect a response to changes in internal osmotic balance. Neutral amino acids such as alanine, valine, proline, and glycine are known to be subject to efflux to combat the effects of hypotonic swelling (39). The observed reductions in the levels of the amino acids phenylalanine and tyrosine, which can be catabolized to produce both DOPA and pyomelanin, likely explain the reduced melanization observed in the Δ*simA* mutant at 37°C on BHI medium and the altered colony morphology seen under conditions of growth with tyrosine as the sole nitrogen source (Fig. S2).

The Δ*simA* mutant also exhibited a significant decrease in the amount of *N*-acetylglucosamine (GlcNAc), which could reflect changes in the synthesis of the sugar nucleotide UDP-GlcNAc, the main sugar donor for cell wall chitin biosynthesis (Table S4). Decreased synthesis of chitin in Δ*simA* cells *in vivo* at 37°C correlates with reduced labeling with calcofluor, which bound to chitin in fungal cell walls (Fig. 3B), and with the sensitivity to calcofluor displayed in plate tests at 37°C (Fig. 6).

A significant increase in levels of *myo*-inositol was also observed in the Δ*simA* mutant (Fig. 9), which is the precursor for synthesis of the bulk phospholipid phosphatidylinositol, inositolphosphoceramide, GPI glycolipids, and complex phosphoinositides and derived inositol phosphates.

### Ergosterol distribution is not affected by deletion of *simA.*

Ergosterol is an important component of the fungal cell membrane and the primary target of many antimycotic drugs. Ergosterol biosynthesis involves the activity of a number of cytochrome P450s (e.g., *S. cerevisiae* Erg11p and Erg5p), none of which are orthologous to that encoded by *simA* (19, 40). Distribution of ergosterol was qualitatively assessed using the fluorescent polyene macrolide filipin stain that specifically intercalates into sterol-rich membranes, allowing visualization of cellular sterols (41, 42). In the wild type, ergosterol concentrations are observed at the growing cell apex and at the plasma membrane, including at septa. Ergosterol distributions and levels were indistinguishable between the wild-type and Δ*simA* strains using filipin staining at 25°C and 37°C (not shown). The sterol levels in mid-log-phase yeast cells of the wild-type, Δ*simA*, and Δ*simA simA*^*+*^ strains grown at 37°C were measured (Materials and Methods). Sterol abundance was determined for each strain, and paired Student’s *t* tests were performed to identify sterols whose abundance significantly differed between strains (*P* value < 0.05) (Table S5). Metabolites which significantly changed in abundance were identified where possible by comparison to mass spectral libraries (Tables S5). As expected, levels of ergosterol did not significantly differ between the wild-type, Δ*simA*, and Δ*simA simA*^*+*^ strains.

10.1128/mSphere.00056-18.7 TABLE S5  Sterols showing significant differences between the wild-type and Δ*simA* mutant strains in the levels of mid-log-phase yeast cells. Download TABLE S5, DOC file, 0.1 MB. Copyright © 2018 Boyce et al. 2018 Boyce et al. This content is distributed under the terms of the Creative Commons Attribution 4.0 International license.

## DISCUSSION

The yeast-specific cytochrome P450 encoded by *simA* has no readily predictable function due to its lack of proximity to genes encoding PKS or NRPS or clear secondary metabolite clusters. However, the generation of a gene deletion strain has uncovered an essential role for *simA* during both growth and survival in macrophages. In macrophages, the Δ*simA* strain exhibits reduced conidial germination and poorer subsequent yeast proliferation, suggesting that the enzymatic product of *simA* is required to facilitate (to stimulate or protect) fungal growth *in vivo*. Intracellular Δ*simA* yeast cells exhibited a marked defect in cell wall integrity, as shown by loss of calcofluor staining, and were subsequently lysed and degraded in intracellular vacuoles. Fungal lysis is not a consequence of decreased germination *per se*, as other *T. marneffei* mutants which exhibit reduced germination in macrophages do not undergo similar lysis (43–45). Both the wild-type strain and the Δ*simA* mutant were retained within prephagolysosomal compartments, suggesting that the loss of viability of the mutant was not due to differential targeting to the mature lysosome. Metabolite profiling studies indicated that loss of SimA results in loss of metabolic activity *in vivo*, with decreased production of pyruvate and a concomitant increase in synthesis of glycerol and other osmolytes to cope with osmotic stress. The decrease in the abundance of neutral amino acids, especially alanine, and the increase in the abundance of the alanine analogue aminoisobutyric acid also suggest that the Δ*simA* mutant was experiencing high levels of osmotic stress. The efflux of the neutral amino acids alanine, valine, proline, and glycine and the influx of aminoisobutyric acid have been shown to be important mechanisms in combating osmotic stress in other intracellular pathogens, including the parasites *Leishmania major*, *Crithidia luciliae*, and *Giardia intestinalis* (46–48).

Synthesis and retention of the osmolyte glycerol are mostly governed by the high-osmolarity glycerol (HOG) signaling pathway (reviewed in reference 36). The central core of the pathway is a mitogen-activated protein kinase (MAPK) cascade, and the pathway culminates in the activation of the MAPK Hog1p. Phosphorylated Hog1p interacts with a number of transcription factors responsible for the induction of genes required for the response to osmotic stress, including those required for the synthesis of glycerols such as glycerol-3-phosphate dehydrogenase and glycerol-3-phosphatases (36). There is evidence that *S. cerevisiae* Hog1p may also directly control metabolic flux in response to stress, as Hog1p regulates Pfk2p, the 6-phosphofructo-2-kinase which controls the levels of fructose-2-6-bisphosphate, a key activator of glycolysis (49). It remains to be determined whether changes in glycerol synthesis/activation of the HOG pathway occur in response to defects in cell wall synthesis, with possible loss of membrane integrity, or whether dysregulation of this pathway leads to osmotic stress and breakdown of the cell wall. Regardless, fungal cell wall chitin is used to mask or obscure cell wall components detected by the host, and a reduction in chitin levels would therefore result in increased recognition and degradation by the macrophage (12, 50, 51).

The *simA* mutant shows many phenotypic similarities to the *Aspergillus fumigatus* Δ*ppoC* mutant, including increased tolerance of H_2_O_2_, decreased germination of conidia, and increased killing by macrophages (6, 9). *A. fumigatus ppoC* is required for the production of the oxylipin prostaglandin E2 (PGE_2_), which has been shown to influence the microbe-host interaction during infection (6–9). Fungal oxylipins are thought to modulate host immune functions due to their similarities to the host eicosanoids, which act as short-range hormones involved in immune responses such as inflammation (8, 52). Like mammalian PGE_2_, fungal PGE_2_ derived from *Cryptococcus neoformans* and *Candida albicans* has been shown to reduce lymphocyte proliferation and to downregulate the production of the inflammatory cytokines tumor necrosis factor alpha (TNF-α) and interleukin-8 (IL-8) while conversely increasing the production of the anti-inflammatory cytokine interleukin-10 (IL-10) (8, 52). The addition of host- or fungus-derived oxylipin PGE_2_ also enhances *C. albicans* germ tube formation (8, 53). Given that the Δ*ppoC* mutant also exhibits decreased germination of conidia *in vivo*, oxylipins appear to be required to stimulate growth during macrophage infection. In mammals, the biosynthesis of oxylipins is initiated by dioxygenases or CYPs. Currently, very little is known about the biochemistry of oxylipin production in eukaryotic microbes as they are difficult to investigate due to a wide range of stereochemical structures and the labile nature of these compounds (reviewed in reference 54). It is tempting to speculate that, like *ppoC*, *simA* may be involved in the production of an oxylipin, a theory which is also supported by the increase in the level of *myo*-inositol (a precursor of oxylipins) displayed in the Δ*simA* mutant. However, the enzymatic product of SimA remains unidentified at this stage. It will be of great interest to elucidate the reaction catalyzed by SimA that allows *T. marneffei* to occupy the specific environmental niche of a macrophage within the host.

## MATERIALS AND METHODS

### Expression analysis.

RNA from the FRR2161 type strain (the wild-type strain) was isolated from vegetative hyphal cells grown at 25°C for 2 days in liquid medium, from asexually developing cultures grown on solid medium at 25°C for 7 days, and from yeast cells grown at 37°C for 6 days in liquid medium. RNA was isolated from yeast cells derived either from LPS-activated J774 murine macrophages infected with wild-type conidia at 24 h postinfection or from yeast cells incubated in macrophage growth media for 24 h. Macrophages were infected as described in the "*In vivo* macrophage assay" section below. RNA was extracted using TRIzol reagent (Invitrogen) and an MP FastPrep-24 bead beater according to the manufacturer’s instructions. RNA was DNase treated (Promega) prior to RT-PCR analysis, and a synthesis control assay lacking cDNA was performed to ensure that no DNA contamination was present. Increasing numbers of cycles were used to ensure that the amplification was in the exponential phase, and the *benA* gene was used as a loading control. Expression of *simA* was determined by RT-PCR (Invitrogen Superscript III One-Step RT-PCR with platinum *Taq*) using primers *simA-*DD3 (5′-ATCCATCCCCCGTGAAGC-3′) and *simA-*DD4 (5′-GCCGACACGAAGTGATCC-3′). Band intensity was quantitated in Photoshop, and relative intensity values were calculated using the *benA* loading controls.

### Molecular techniques.

*T. marneffei* genomic DNA was isolated as previously described (55). Southern blotting was performed with an Amersham Hybond N+ membrane according to the manufacturer’s instructions. Filters were hybridized using [α-^32^P]dATP-labeled probes by standard methods (56).

The *simA* coding region, 1,029 bp of the promoter, and 1,348 bp of the 3′ region were amplified with primers CC76 (5′-GAGTGGTCGAGTCGTGCG-3′) and CC77 (5′-CCGCAGAAACTCCCAAAC-3′). The 4,097-bp PCR product was cloned into pGEM-T Easy (Promega), generating pKB7097. A *simA* split-marker deletion construct was generated by overlap PCR as follows. The 5′ promoter region of *simA* was amplified using CC76 (5′-GAGTGGTCGAGTCGTGCG-3′) and CC78 (5′-AAGGGTGAACACTATCCGAAGGGGGCAAAATATCC-3′). The 3′ *simA* region was amplified using CC77 (5′-CCGCAGAAACTCCCAAAC-3′) and CC80 (5′-GATGAGTGGCAGGGGGCTCTGGATCTTTCAAGGGC-3′). The 5′ region of the *pyrG* selectable marker was amplified using CC79 (5′-GGATATTTTGCCCCCTTCGGATAGTGTTCACCCTT-3′) and AA52 (5′-CTTATCGGGCCGGAGCA-3′). The 3′ region of *pyrG* was amplified using CC81 (5′-GCCCTTGAAAGATCCAGAGCCCCCTGCCACTCATC-3′) and AA53 (5′-ATCCTCGCTCTCCTCTTTCT-3′). PCR products from the CC76-and-CC78 reaction and the CC79-and-AA52 reaction were mixed and amplified using overlap PCR with primers CC76 and AA52. This PCR product was cloned into pGEM-T Easy to generate pKB7098. The PCR products from the CC77 and CC80 reaction and the CC81 and AA53 reaction were also joined and amplified by overlap PCR with the primers CC77 and AA53. This PCR product was cloned into pGEM-T Easy to generate pKB7099. During fungal transformation of the linear fragments from pKB7098 and pKB7099, three recombination events allowed homologous integration at the *simA* locus, the generation of a functional *pyrG* gene, and deletion of the entire *simA* coding region (and 37 bp of the 5′ region and 40 bp of the 3′ region). The Δ*simA* complementation construct (pKB7784) was generated by cloning a 4-kb SpeI/NotI fragment from pKB7097 into a vector containing the *barA* selectable marker (pSM6355) digested with SpeI/NotI. A *simA* HA-tagged localization construct (pKB7798) was generated by insertion of an EcoICRI/XhoI fragment containing a triple-HA tag from pKB7798 into a *simA* inverse PCR product generated with primers MM80 (5′-AAACTCGAGAATGTTCTCATCCGGCGG-3′) and MM81 (5′-AATAGGCCTTGGCCCAGTCTTTGCCGA-3′) digested with StuI/XhoI.

### Fungal strains and media.

Transformation was performed as previously described (55). Strains FRR2161, Δ*pkuA*::*pyrG*^*+*^ (G681), Δ*pkuA pyrG*^*−*^ (G526), and Δ*ligD pyrG*^*−*^ have been previously described (33, 55).

The Δ*simA* strain (Δ*simA*::*pyrG*^*+*^ Δ*pkuA niaD pyrGareA*^Δ*DBD*^) (G559) (where "DBD" represents "DNA binding domain") was generated by transformation of strain G526 (Δ*pkuA niaD pyrGareA*^Δ*DBD*^) with PCR products amplified from pKB7098 (primers CC76 and AA52) and pKB7099 (primers CC77 and AA53) and selection for *pyrG*^+^. Deletion of *simA* was confirmed by genomic Southern analysis. The G559 strain was made *areA*^*+*^ by transformation with pHS6104 to generate G891. The Δ*simA* mutation was complemented by transformation of the Δ*simA* (G891) strain with pKB7784 and selection for glufosinate resistance to generate the Δ*simA simA*^*+*^ strain (G893). A Δ*simA* strain (G1023) was also generated in strain G816 (Δ*ligD niaD pyrG*). The *simA*::*HA* strain (G1017) was generated by cotransformation of strain G147 with pKB7798 and pAB4626 (*pyrG*^+^).

Strains were grown at 25°C on *A. nidulans* minimal medium (ANM) supplemented with 1% glucose and 10 mM ammonium sulfate [(NH_4_)_2_SO_4_] as a sole nitrogen source (57). Strains were grown at 37°C on brain heart infusion (BHI) medium or on synthetic minimal medium (SD medium) supplemented with 10 mM (NH_4_)_2_SO_4_ (58). To test pyomelanin production, strains were grown on *A. nidulans* minimal medium (ANM) supplemented with 1% glucose and 10 mM tyrosine. L-DOPA medium was prepared as previously described (34). To test sensitivity to salt stress and to osmotic and oxidative stress, strains were grown for 10 days at 25°C or 6 days at 37°C on ANM media (25°C) or SD media (37°C) plus 10 mM (NH_4_)_2_SO_4_ supplemented with 0.3 M or 0.6 M NaCl; 0.5 M or 1 M sorbitol; 0.5 mM, 1 mM, 2 mM, or 10 mM H_2_O_2_; or 0.5, 1, 5, or 10 mM NO_2_. For cell wall tests, strains were grown for 10 days at 25°C or for 6 days at 37°C on ANM plus 10 mM (NH_4_)_2_SO_4_ plus 2.5, 5, 10, or 15 μM Congo red or 10, 15, 20, or 30 μg/ml calcofluor white. At 25°C, stress plates were inoculated with a 10-μl drop of a 1 × 10^5^ conidia/ml suspension. At 37°C, stress plates were inoculated with 10-μl drops of 10-fold serial dilutions of a 1 × 10^7^ conidia/ml suspension.

### *In vivo* macrophage assay.

J774 murine macrophages (1 × 10^5^) were seeded into each well of a 6-well microtiter tray containing one sterile coverslip and 2 ml of complete Dulbecco’s modified Eagle medium (complete DMEM; DMEM, 10% fetal bovine serum, 8 mM l-glutamine, and penicillin-streptomycin). Macrophages were incubated at 37°C for 24 h before activation with 0.1 µg·ml^−1^ lipopolysaccharide (LPS) from *Escherichia coli* (Sigma). Macrophages were incubated a further 24 h at 37°C and washed in phosphate-buffered saline (PBS), and 2 ml of complete DMEM containing 1 × 10^6^ conidia was added. A control experiment lacking conidia was also performed. Macrophages were incubated for 2 h at 37°C (to allow conidia to be phagocytosed), washed once in PBS (to remove nonphagocytosed conidia), and either fixed or incubated for a further 24 or 48 h at 37°C. Macrophages were fixed in 4% paraformaldehyde and stained with 1 mg·ml^−1^ fluorescent brightener 28 (calcofluor [CAL]) to observe fungal cell walls. The numbers of ungerminated conidia, germlings, or yeast cells were recorded in a population of approximately 100 in three independent experiments. The numbers of calcofluor-staining cells in a population of approximately 100 in three independent experiments were recorded. Means and standard errors of the mean values were calculated using GraphPad Prism3.

### Microscopy.

*T. marneffei* strains were grown on slides covered with a thin layer of solid medium, with one end resting in liquid medium (55). Wild-type, Δ*simA*, and Δ*simA simA*^*+*^ strains were grown on either 0.1% or 1% ANM medium supplemented with (NH_4_)_2_SO_4_ for 2 days (1%) or 4 days (0.1%). Strains were grown for 6 days at 37°C on BHI or SD medium supplemented with (NH_4_)_2_SO_4_. Immunofluorescence microscopy was performed for examination of the early endosomes and lysosome with either a mouse monoclonal anti-LAMP1 primary antibody (Santa Cruz Biotechnology) or a mouse monoclonal anti-cathepsin D primary antibody (Abcam, Inc.) and an Alexa 488 rabbit anti-mouse secondary antibody (Molecular Probes). No primary antibody controls were performed to confirm the specificity of the antibodies.

Slides were examined using differential interference contrast (DIC) and epifluorescence optics for cell wall staining with calcofluor or for nucleus staining with Hoescht 33258 and viewed on a Reichart Jung Polyvar II microscope. Images were captured using a Spot charge-coupled-device (CCD) camera (Diagnostic Instruments, Inc.) and processed in Adobe Photoshop. For transmission electron microscopy (TEM), agar cubes containing the fungal biomass or trypsin-treated infected macrophages were fixed with 2.5% glutaraldehyde–PBS buffer for 2 h, washed three times in PBS, and postfixed with 1% osmium tetroxide for 2 h. Samples were then washed three times in PBS and subjected to ethanol dehydration by washes performed with increasing concentrations of ethanol. Samples were embedded in white resin, and thin sections were examined with a Philips CM120 BioTWIN transmission electron microscope.

### Metabolomic analysis.

Wild-type, Δ*simA*, and Δ*simA simA*^*+*^ strains were cultured in brain heart infusion (BHI) medium for 4 days at 37°C. A 10-ml volume of this yeast culture was transferred to a fresh BHI flask, and mid-log-phase yeast cells were harvested after 20 h (59). Separate polar and sterol metabolomic analyses were performed on 4 biological repeats for each strain, with 4 technical repeats. Yeast cells (1 × 10^8^) were metabolically quenched by rapid filtration on sterilized filter disks using a suction apparatus and were then dried (59). The filter disks were divided into four replicates (2.5 × 10^7^) and then lysed in 3:1 (vol/vol) methanol/Milli-Q water (600 µl) using a freeze/thaw method, where samples were cycled (10 times for 30 s each time) in liquid N2, followed by a dry ice/ethanol bath. Metabolites were further extracted by addition of chloroform (1:3:1 [vol/vol/vol] chloroform/methanol/water; 150 µl). Samples were centrifuged (4°C, 14,000 rpm, 5 min) to pellet cell debris and precipitated macromolecules, and the resultant supernatant was transferred to a fresh microcentrifuge tube. Samples were biphasic and were partitioned by the further addition of 300 µl Milli-Q water (1:3:3 [vol/vol/vol] chloroform/methanol/water), and the upper aqueous methanol/water phase containing polar metabolites and lower chloroform phase containing sterols were separately analyzed by GC-MS.

### Sterol analysis.

Chloroform phases were evaporated to dryness *in vacuo*, and trimethylsilyl (TMS)-derivatized {BSTFA [N,O-bis(trimethylsilyl)trifluoroacetamide] plus 1% TMCS (trimethylchlorosilane), 40 µl, 37°C, 60 min} samples were analyzed by GC-MS. Briefly, 1 µl of derivatized sample was injected into a hot inlet (250°C) and separated on an Agilent VF-5ms column (30 m by 0.25-mm inner diameter [i.d.] by 0.25 µM film thickness) using an Agilent 7890 GC system coupled to a 5975C mass-selective detector. The GC oven temperature ramp was started at 150°C, was held there for 1 min, and then was raised 25°C/min to 285°C, held for 3.5 min, and finally raised 3°C/min to 315°C. The transfer line was set to 280°C, and the mass spectrometer set to scan 50 to 600 m/z at 2.66 scans/s. Pooled biological quality control (PBQC) samples were run throughout the sequence for quality assurance and data normalization purposes. Samples and quality controls were aligned in an untargeted manner using PyMS analysis software (60), producing a data matrix of 176 aligned peaks.

### Polar metabolite analysis.

Aqueous phases were evaporated to dryness *in vacuo*, subjected to methoximation and TMS derivatization, and then analyzed using the MA_25C GC-MS method (25°C/min oven ramp) (61). The instrument and column were used as described above. Pooled biological quality control (PBQC) samples were run throughout the sequence for quality assurance and data normalization purposes. Samples and quality controls were aligned in an untargeted manner using PyMS analysis software (60), producing a data matrix of 466 aligned peaks.

### Statistical analyses.

The mean, median, and standard deviation data of metabolite intensities for each sample were plotted, and the results were compared to internal standard intensities. In some instances, the internal standard intensity did not correlate with the mean sample value, and so a median normalization of the data was used, prior to performing multivariate and univariate statistical analyses. Univariate analysis was applied to the data, with paired Student’s *t* tests performed on all combinations of sample groups (*P* value < 0.05). Further false-discovery-rate analysis was performed using a Benjamini-Hochberg (BH) adjustment (BH-adjusted *P* value < 0.05). Lists were sorted into lowest BH-adjusted *P* values and highest fold changes. Significantly changing metabolites were identified where possible by comparison to mass spectral libraries.

### Data availability.

All data sets are available in the supplemental material.
