# Supplementary material for: Talaromyces marneffei simA Encodes a Fungal Cytochrome P450 Essential for Survival in Macrophages
Source: mSphere. 2018 Mar 21;3(2):e00056-18. doi: 10.1128/mSphere.00056-18 (PMC5863032; doi:10.1128/mSphere.00056-18)
Supplement: TABLE S1 [file sph002182498st1.doc]

**Supplementary Table 1. *T. marneffei* Cytochrome P450s**

| **PMAA number** | **Protein accession number** | **Nelson’s best hit**  **CYP classification** | **PMAA number** | **Protein accession number** | **Nelson’s best hit**  **CYP classification** |
| --- | --- | --- | --- | --- | --- |
| **PMAA_069510** | [EEA25858.1](http://p450.riceblast.snu.ac.kr/class.php?a=dv_sequence&id=12010&spe_id=7910&ref_id=3409) | CYP51F1P | **PMAA_101770** | [EEA23592.1](http://p450.riceblast.snu.ac.kr/class.php?a=dv_sequence&id=12052&spe_id=7910&ref_id=3409) | CYP5041A2 |
| **PMAA_054960** | EEA21701.1 | CYP61A1 | **PMAA_001070** | [EEA19307.1](http://p450.riceblast.snu.ac.kr/class.php?a=dv_sequence&id=12075&spe_id=7910&ref_id=3409) | CYP5195B1 |
| **PMAA_001660** | [EEA19371.1](http://p450.riceblast.snu.ac.kr/class.php?a=dv_sequence&id=12076&spe_id=7910&ref_id=3409) | CYP53A18 | **PMAA_062960** | [EEA25175.1](http://p450.riceblast.snu.ac.kr/class.php?a=dv_sequence&id=12003&spe_id=7910&ref_id=3409) | CYP5077B1 |
| **PMAA_002780** | [EEA19486.1](http://p450.riceblast.snu.ac.kr/class.php?a=dv_sequence&id=12077&spe_id=7910&ref_id=3409) | CYP578B2 | **PMAA_101680** | [EEA23583.1](http://p450.riceblast.snu.ac.kr/class.php?a=dv_sequence&id=12051&spe_id=7910&ref_id=3409) | CYP5116A1 |
| **PMAA_076430** | EEA26582.1 | CYP6001C16 | **PMAA_020360** | [EEA27145.1](http://p450.riceblast.snu.ac.kr/class.php?a=dv_sequence&id=11975&spe_id=7910&ref_id=3409) | CYP539J1 |
| **PMAA_087970** | [EEA24822.1](http://p450.riceblast.snu.ac.kr/class.php?a=dv_sequence&id=12025&spe_id=7910&ref_id=3409) | CYP504A7 | **PMAA_031760** | EEA28362.1 | CYP548A2 |
| **PMAA_080510** | [EEA24033.1](http://p450.riceblast.snu.ac.kr/class.php?a=dv_sequence&id=12021&spe_id=7910&ref_id=3409) | CYP504B8 | **PMAA_038590** | EEA29077.1 | CYP620E3 |
| **PMAA_096640** | [EEA23068.1](http://p450.riceblast.snu.ac.kr/class.php?a=dv_sequence&id=12036&spe_id=7910&ref_id=3409) | CYP657B3 | **PMAA_065360** | [EEA25426.1](http://p450.riceblast.snu.ac.kr/class.php?a=dv_sequence&id=12007&spe_id=7910&ref_id=3409) | CYP5068C1 |
| **PMAA_018640** | [EEA26956.1](http://p450.riceblast.snu.ac.kr/class.php?a=dv_sequence&id=11973&spe_id=7910&ref_id=3409) | CYP548D7 | **PMAA_054540** | [EEA21658.1](http://p450.riceblast.snu.ac.kr/class.php?a=dv_sequence&id=12059&spe_id=7910&ref_id=3409) | CYP578C2 |
| **PMAA_034820** | [EEA28684.1](http://p450.riceblast.snu.ac.kr/class.php?a=dv_sequence&id=11996&spe_id=7910&ref_id=3409) | CYP65Z2 | **PMAA_071760** | EEA26095.1 | CYP5076A3 |
| **PMAA_034710** | [EEA28673.1](http://p450.riceblast.snu.ac.kr/class.php?a=dv_sequence&id=11994&spe_id=7910&ref_id=3409) | CYP530A5 | **PMAA_014830** | [EEA19223.1](http://p450.riceblast.snu.ac.kr/class.php?a=dv_sequence&id=12090&spe_id=7910&ref_id=3409) | CYP682J2 |
| **PMAA_034830** | [EEA28685.1](http://p450.riceblast.snu.ac.kr/class.php?a=dv_sequence&id=11997&spe_id=7910&ref_id=3409) | CYP551A2 | **PMAA_010260** | [EEA18744.1](http://p450.riceblast.snu.ac.kr/class.php?a=dv_sequence&id=12087&spe_id=7910&ref_id=3409) | CYP532B4 |
| **PMAA_034760** | [EEA28678.1](http://p450.riceblast.snu.ac.kr/class.php?a=dv_sequence&id=11995&spe_id=7910&ref_id=3409) | CYP5042B1P | **PMAA_050760** | [EEA21258.1](http://p450.riceblast.snu.ac.kr/class.php?a=dv_sequence&id=12058&spe_id=7910&ref_id=3409) | CYP602B3 |
| **PMAA_031320** | [EEA28318.1](http://p450.riceblast.snu.ac.kr/class.php?a=dv_sequence&id=11985&spe_id=7910&ref_id=3409) | CYP5082A3 | **PMAA_073050** | [EEA26229.1](http://p450.riceblast.snu.ac.kr/class.php?a=dv_sequence&id=12015&spe_id=7910&ref_id=3409) | CYP539D10 |
| **PMAA_031360** | [EEA28322.1](http://p450.riceblast.snu.ac.kr/class.php?a=dv_sequence&id=11986&spe_id=7910&ref_id=3409) | CYP5093A1 | **PMAA_093890** | [EEA22775.1](http://p450.riceblast.snu.ac.kr/class.php?a=dv_sequence&id=12035&spe_id=7910&ref_id=3409) | CYP52T1 |
| **PMAA_063570** | [EEA25395.1](http://p450.riceblast.snu.ac.kr/class.php?a=dv_sequence&id=12006&spe_id=7910&ref_id=3409) | CYP584E8 | **PMAA_059650** | [EEA22186.1](http://p450.riceblast.snu.ac.kr/class.php?a=dv_sequence&id=12061&spe_id=7910&ref_id=3409) | CYP52H4 |
| **PMAA_063650** | [EEA25248.1](http://p450.riceblast.snu.ac.kr/class.php?a=dv_sequence&id=12005&spe_id=7910&ref_id=3409) | CYP62A1 | **PMAA_099260** | [EEA23338.1](http://p450.riceblast.snu.ac.kr/class.php?a=dv_sequence&id=12044&spe_id=7910&ref_id=3409) | CYP663B4 |
| **PMAA_043610** | [EEA20527.1](http://p450.riceblast.snu.ac.kr/class.php?a=dv_sequence&id=12066&spe_id=7910&ref_id=3409) | CYP54C2 | **PMAA_102120** | [EEA23628.1](http://p450.riceblast.snu.ac.kr/class.php?a=dv_sequence&id=12054&spe_id=7910&ref_id=3409) | CYP58M2 |
| **PMAA_043600** | [EEA20526.1](http://p450.riceblast.snu.ac.kr/class.php?a=dv_sequence&id=12065&spe_id=7910&ref_id=3409) | CYP570H1 | **PMAA_022530** | [EEA27374.1](http://p450.riceblast.snu.ac.kr/class.php?a=dv_sequence&id=11976&spe_id=7910&ref_id=3409) | CYP58M2 |
| **PMAA_043670** | [EEA20533.1](http://p450.riceblast.snu.ac.kr/class.php?a=dv_sequence&id=12068&spe_id=7910&ref_id=3409) | CYP660A2 | **PMAA_068620** | [EEA25768.1](http://p450.riceblast.snu.ac.kr/class.php?a=dv_sequence&id=12009&spe_id=7910&ref_id=3409) | CYP540B15 |
| **PMAA_043550** | [EEA20521.1](http://p450.riceblast.snu.ac.kr/class.php?a=dv_sequence&id=12064&spe_id=7910&ref_id=3409) | CYP503B4 | **PMAA_037350** | [EEA28949.1](http://p450.riceblast.snu.ac.kr/class.php?a=dv_sequence&id=11999&spe_id=7910&ref_id=3409) | CYP682G1 |
| **PMAA_043630** | [EEA20529.1](http://p450.riceblast.snu.ac.kr/class.php?a=dv_sequence&id=12067&spe_id=7910&ref_id=3409) | CYP660B1 | **PMAA_003930** | [EEA19609.1](http://p450.riceblast.snu.ac.kr/class.php?a=dv_sequence&id=12078&spe_id=7910&ref_id=3409) | CYP65U2 |
| **PMAA_050330** | [EEA21215.1](http://p450.riceblast.snu.ac.kr/class.php?a=dv_sequence&id=12055&spe_id=7910&ref_id=3409) | CYP584E8 | **PMAA_009620** | [EEA18679.1](http://p450.riceblast.snu.ac.kr/class.php?a=dv_sequence&id=12086&spe_id=7910&ref_id=3409) | CYP547C2 |
| **PMAA_050350** | [EEA21217.1](http://p450.riceblast.snu.ac.kr/class.php?a=dv_sequence&id=12056&spe_id=7910&ref_id=3409) | CYP617A2 | **PMAA_098060** | [EEA23218.1](http://p450.riceblast.snu.ac.kr/class.php?a=dv_sequence&id=12040&spe_id=7910&ref_id=3409) | CYP537B2 |
| **PMAA_050410** | [EEA21223.1](http://p450.riceblast.snu.ac.kr/class.php?a=dv_sequence&id=12057&spe_id=7910&ref_id=3409) | CYP585A1 | **PMAA_007670** | [EEA20000.1](http://p450.riceblast.snu.ac.kr/class.php?a=dv_sequence&id=12082&spe_id=7910&ref_id=3409) | CYP5054A1 |
| **PMAA_088100** | [EEA24836.1](http://p450.riceblast.snu.ac.kr/class.php?a=dv_sequence&id=12026&spe_id=7910&ref_id=3409) | CYP68L1 | **PMAA_014720** | [EEA19212.1](http://p450.riceblast.snu.ac.kr/class.php?a=dv_sequence&id=12089&spe_id=7910&ref_id=3409) | CYP628F1 |
| **PMAA_088180** | [EEA24844.1](http://p450.riceblast.snu.ac.kr/class.php?a=dv_sequence&id=12028&spe_id=7910&ref_id=3409) | CYP584E2 | **PMAA_015690** | [EEA26647.1](http://p450.riceblast.snu.ac.kr/class.php?a=dv_sequence&id=11971&spe_id=7910&ref_id=3409) | CYP65AB2 |
| **PMAA_088170** | EEA24843.1 | CYP58M2 | **PMAA_023420** | [EEA27467.1](http://p450.riceblast.snu.ac.kr/class.php?a=dv_sequence&id=11977&spe_id=7910&ref_id=3409) | CYP5080 |
| **PMAA_031520** | [EEA28338.1](http://p450.riceblast.snu.ac.kr/class.php?a=dv_sequence&id=11987&spe_id=7910&ref_id=3409) | CYP5282C1 | **PMAA_026950** | [EEA27853.1](http://p450.riceblast.snu.ac.kr/class.php?a=dv_sequence&id=11982&spe_id=7910&ref_id=3409) | CYP65B2 |
| **PMAA_031530** | EEA28339.1 | CYP584 | **PMAA_029540** | [EEA28136.1](http://p450.riceblast.snu.ac.kr/class.php?a=dv_sequence&id=11983&spe_id=7910&ref_id=3409) | CYP5090A1 |
| **PMAA_025880** | [EEA27738.1](http://p450.riceblast.snu.ac.kr/class.php?a=dv_sequence&id=11979&spe_id=7910&ref_id=3409) | CYP5171A1 | **PMAA_029570** | [EEA28139.1](http://p450.riceblast.snu.ac.kr/class.php?a=dv_sequence&id=11984&spe_id=7910&ref_id=3409) | CYP5100A1 |
| **PMAA_025930** | [EEA27743.1](http://p450.riceblast.snu.ac.kr/class.php?a=dv_sequence&id=11980&spe_id=7910&ref_id=3409) | CYP682H1 | **PMAA_039010** | [EEA20034.1](http://p450.riceblast.snu.ac.kr/class.php?a=dv_sequence&id=12062&spe_id=7910&ref_id=3409) | CYP559F1 |
| **PMAA_098630** | [EEA23273.1](http://p450.riceblast.snu.ac.kr/class.php?a=dv_sequence&id=12041&spe_id=7910&ref_id=3409) | CYP504E2 | **PMAA_050000** | [EEA21202.1](http://p450.riceblast.snu.ac.kr/class.php?a=dv_sequence&id=12074&spe_id=7910&ref_id=3409) | CYP5050A1 |
| **PMAA_098650** | [PEEA23275.1](http://p450.riceblast.snu.ac.kr/class.php?a=dv_sequence&id=12042&spe_id=7910&ref_id=3409) | CYP684A4 | **PMAA_061990** | [EEA25068.1](http://p450.riceblast.snu.ac.kr/class.php?a=dv_sequence&id=12002&spe_id=7910&ref_id=3409) | CYP559 |
| **PMAA_085890** | [EEA24596.1](http://p450.riceblast.snu.ac.kr/class.php?a=dv_sequence&id=12023&spe_id=7910&ref_id=3409) | CYP504E1 | **PMAA_076030** | [EEA26536.1](http://p450.riceblast.snu.ac.kr/class.php?a=dv_sequence&id=12016&spe_id=7910&ref_id=3409) | CYP52G10 |
| **PMAA_085840** | [EEA24591.1](http://p450.riceblast.snu.ac.kr/class.php?a=dv_sequence&id=12022&spe_id=7910&ref_id=3409) | CYP5128A2 | **PMAA_077100** | [EEA23677.1](http://p450.riceblast.snu.ac.kr/class.php?a=dv_sequence&id=12019&spe_id=7910&ref_id=3409) | CYP595B3 |
| **PMAA_007450** | [EEA19978.1](http://p450.riceblast.snu.ac.kr/class.php?a=dv_sequence&id=12080&spe_id=7910&ref_id=3409) | CYP65AF1 | **PMAA_087090** | [EEA24730.1](http://p450.riceblast.snu.ac.kr/class.php?a=dv_sequence&id=12024&spe_id=7910&ref_id=3409) | CYP65Z2 |
| **PMAA_007490** | [EEA19982.1](http://p450.riceblast.snu.ac.kr/class.php?a=dv_sequence&id=12081&spe_id=7910&ref_id=3409) | CYP575A1 | **PMAA_089450** | [EEA24974.1](http://p450.riceblast.snu.ac.kr/class.php?a=dv_sequence&id=12029&spe_id=7910&ref_id=3409) | CYP5093A4 |
| **PMAA_007440** | [EEA19977.1](http://p450.riceblast.snu.ac.kr/class.php?a=dv_sequence&id=12079&spe_id=7910&ref_id=3409) | CYP617D6 | **PMAA_101130** | [EEA23526.1](http://p450.riceblast.snu.ac.kr/class.php?a=dv_sequence&id=12050&spe_id=7910&ref_id=3409) | CYP5043C1 |
| **PMAA_097910** | [EEA23202.1](http://p450.riceblast.snu.ac.kr/class.php?a=dv_sequence&id=12039&spe_id=7910&ref_id=3409) | CYP5075A2 | **PMAA_101970** | [EEA23614.1](http://p450.riceblast.snu.ac.kr/class.php?a=dv_sequence&id=12053&spe_id=7910&ref_id=3409) | CYP533B1 |
| **PMAA_097900** | [EEA23201.1](http://p450.riceblast.snu.ac.kr/class.php?a=dv_sequence&id=12038&spe_id=7910&ref_id=3409) | CYP5090A1 | **PMAA_026510** | [EEA27807.1](http://p450.riceblast.snu.ac.kr/class.php?a=dv_sequence&id=11981&spe_id=7910&ref_id=3409) | CYP5050A1 |
| **PMAA_097880** | [EEA23199.1](http://p450.riceblast.snu.ac.kr/class.php?a=dv_sequence&id=12037&spe_id=7910&ref_id=3409) | CYP606B2 | **PMAA_024240** | [EEA27553.1](http://p450.riceblast.snu.ac.kr/class.php?a=dv_sequence&id=11978&spe_id=7910&ref_id=3409) | CYP53A23 |
| **PMAA_032120** | [EEA28399.1](http://p450.riceblast.snu.ac.kr/class.php?a=dv_sequence&id=11990&spe_id=7910&ref_id=3409) | CYP617D8 | **PMAA_015840** | [EEA26662.1](http://p450.riceblast.snu.ac.kr/class.php?a=dv_sequence&id=11972&spe_id=7910&ref_id=3409) | CYP5070A1 |
| **PMAA_032150** | [EEA28402.1](http://p450.riceblast.snu.ac.kr/class.php?a=dv_sequence&id=11991&spe_id=7910&ref_id=3409) | CYP5053C1 | **PMAA_090170** | [EEA25052.1](http://p450.riceblast.snu.ac.kr/class.php?a=dv_sequence&id=12030&spe_id=7910&ref_id=3409) | CYP531C4 |
| **PMAA_092970** | [EEA22677.1](http://p450.riceblast.snu.ac.kr/class.php?a=dv_sequence&id=12033&spe_id=7910&ref_id=3409) | CYP531E1 | **PMAA_048940** | [EEA21088.1](http://p450.riceblast.snu.ac.kr/class.php?a=dv_sequence&id=12072&spe_id=7910&ref_id=3409) | CYP52H4 |
| **PMAA_093020** | [EEA22682.1](http://p450.riceblast.snu.ac.kr/class.php?a=dv_sequence&id=12034&spe_id=7910&ref_id=3409) | CYP5104B2 | **PMAA_013110** | [EEA19044.1](http://p450.riceblast.snu.ac.kr/class.php?a=dv_sequence&id=12088&spe_id=7910&ref_id=3409) | CYP663B1 |
| **PMAA_070450** | [EEA25958.1](http://p450.riceblast.snu.ac.kr/class.php?a=dv_sequence&id=12011&spe_id=7910&ref_id=3409) | CYP619B2 | **PMAA_049850** | [EEA21187.1](http://p450.riceblast.snu.ac.kr/class.php?a=dv_sequence&id=12073&spe_id=7910&ref_id=3409) | CYP684A4 |
| **PMAA_070520** | [EEA25965.1](http://p450.riceblast.snu.ac.kr/class.php?a=dv_sequence&id=12012&spe_id=7910&ref_id=3409) | CYP5107B1 | **PMAA_036040** | EEA28811.1 | CYP52P1 |
| **PMAA_100700** | [EEA23482.1](http://p450.riceblast.snu.ac.kr/class.php?a=dv_sequence&id=12048&spe_id=7910&ref_id=3409) | CYP59D3 | **PMAA_077480** | [EEA23715.1](http://p450.riceblast.snu.ac.kr/class.php?a=dv_sequence&id=12020&spe_id=7910&ref_id=3409) | CYP541B6 |
| **PMAA_100660** | [EEA23478.1](http://p450.riceblast.snu.ac.kr/class.php?a=dv_sequence&id=12047&spe_id=7910&ref_id=3409) | CYP5266B1 | **PMAA_039760** | [EEA20113.1](http://p450.riceblast.snu.ac.kr/class.php?a=dv_sequence&id=12063&spe_id=7910&ref_id=3409) | CYP619B2 |
| **PMAA_033360** | [EEA28529.1](http://p450.riceblast.snu.ac.kr/class.php?a=dv_sequence&id=11993&spe_id=7910&ref_id=3409) | CYP620B2 | **PMAA_098880** | [EEA23298.1](http://p450.riceblast.snu.ac.kr/class.php?a=dv_sequence&id=12043&spe_id=7910&ref_id=3409) | CYP617K1 |
| **PMAA_033280** | [EEA28521.1](http://p450.riceblast.snu.ac.kr/class.php?a=dv_sequence&id=11992&spe_id=7910&ref_id=3409) | CYP614A2 | **PMAA_092140** | [EEA22589.1](http://p450.riceblast.snu.ac.kr/class.php?a=dv_sequence&id=12032&spe_id=7910&ref_id=3409) | CYP58A3 |
| **PMAA_009440** | [EEA18658.1](http://www.ncbi.nlm.nih.gov/protein/210064563) | CYP65B2 | **PMAA_047420** | EEA20930.1 | CYP682H1 |
| **PMAA_009430** | [EEA18657.1](http://www.ncbi.nlm.nih.gov/protein/210064562) | CYP65V6 | **PMAA_038590** | [EEA29076.1](http://p450.riceblast.snu.ac.kr/class.php?a=dv_sequence&id=12000&spe_id=7910&ref_id=3409) | CYP620E3 |
| **PMAA_009420** | [EEA18656.1](http://www.ncbi.nlm.nih.gov/protein/210064561) | CYP638A2 | **PMAA_066300** | [EEA25525.1](http://p450.riceblast.snu.ac.kr/class.php?a=dv_sequence&id=12008&spe_id=7910&ref_id=3409) | CYP5080B3 |
| **PMAA_047040** | [EEA20889.1](http://p450.riceblast.snu.ac.kr/class.php?a=dv_sequence&id=12070&spe_id=7910&ref_id=3409) | CYP577A1 | **PMAA_076600** | [EEA26599.1](http://p450.riceblast.snu.ac.kr/class.php?a=dv_sequence&id=12018&spe_id=7910&ref_id=3409) | CYP660A2 |
| **PMAA_099840** | [EEA23396.1](http://p450.riceblast.snu.ac.kr/class.php?a=dv_sequence&id=12045&spe_id=7910&ref_id=3409) | CYP586E1 | **PMAA_044980** | [EEA20672.1](http://p450.riceblast.snu.ac.kr/class.php?a=dv_sequence&id=12069&spe_id=7910&ref_id=3409) | CYP5076A3 |
| **PMAA_100490** | EEA23461.1 | CYP5134B1P | **PMAA_091100** | [EEA22481.1](http://p450.riceblast.snu.ac.kr/class.php?a=dv_sequence&id=12031&spe_id=7910&ref_id=3409) | CYP628C1 |

* Putative CYP name as Besthit of Nelson’s was >40% identity
