# Supplementary material for: Talaromyces marneffei simA Encodes a Fungal Cytochrome P450 Essential for Survival in Macrophages
Source: mSphere. 2018 Mar 21;3(2):e00056-18. doi: 10.1128/mSphere.00056-18 (PMC5863032; doi:10.1128/mSphere.00056-18)
Supplement: TABLE S2 [file sph002182498st2.doc]

**Supplementary Table 2. *T. marneffei* cytochrome P450s with characterized orthologues in other species**

| **Orthologue** | **PMAA number** | **Protein Accession Number** | **Nelson’s best hit**  **CYP classification** | **Function** |
| --- | --- | --- | --- | --- |
| *ERG11* | PMAA_069510 | [EEA25858.1](http://p450.riceblast.snu.ac.kr/class.php?a=dv_sequence&id=12010&spe_id=7910&ref_id=3409) | CYP51F1P | Ergosterol biosynthesis {Bard, 1993 #1365;Skaggs, 1996 #1369} |
| *ERG5* | PMAA_054960 | EEA21701.1 | CYP61A1 |
| *bzuA* | PMAA_001660 | [EEA19371.1](http://p450.riceblast.snu.ac.kr/class.php?a=dv_sequence&id=12076&spe_id=7910&ref_id=3409) | CYP53A18 | Benzamide utilization {Fraser, 2002 #1349} |
| *ppoC* | PMAA_076430 | EEA26582.1 | CYP6001C16 | Oxylipin biosynthesis {Tsitsigiannis, 2005 #1361;Tsitsigiannis, 2006 #1358} |
| *phacA* | PMAA_087970 | [EEA24822.1](http://p450.riceblast.snu.ac.kr/class.php?a=dv_sequence&id=12025&spe_id=7910&ref_id=3409) | CYP504A7 | Phenylacetate utilization {Mingot, 1999 #1362;Ferrer-Sevillano, 2007 #1363} |
| *phacB* | PMAA_080510 | [EEA24033.1](http://p450.riceblast.snu.ac.kr/class.php?a=dv_sequence&id=12021&spe_id=7910&ref_id=3409) | CYP504B8 |
| *ahbB* | PMAA_096640 | [EEA23068.1](http://p450.riceblast.snu.ac.kr/class.php?a=dv_sequence&id=12036&spe_id=7910&ref_id=3409) | CYP657B3 | Membrane synthesis {Lin, 2004 #453} |
