# Supplementary material for: Talaromyces marneffei simA Encodes a Fungal Cytochrome P450 Essential for Survival in Macrophages
Source: mSphere. 2018 Mar 21;3(2):e00056-18. doi: 10.1128/mSphere.00056-18 (PMC5863032; doi:10.1128/mSphere.00056-18)
Supplement: TABLE S3 [file sph002182498st3.doc]

**Supplementary Table 3. *T. marneffei* cytochrome P450s within gene clusters and those with predicted functions**

| **PMAA number** | **Protein accession number** | **Nelson’s best hit**  **CYP classification** | **Proximity** | **Predicted function** |
| --- | --- | --- | --- | --- |
| PMAA_034820  PMAA_034710  PMAA_034830  PMAA_034760 | [EEA28684.1](http://p450.riceblast.snu.ac.kr/class.php?a=dv_sequence&id=11996&spe_id=7910&ref_id=3409)  [EEA28673.1](http://p450.riceblast.snu.ac.kr/class.php?a=dv_sequence&id=11994&spe_id=7910&ref_id=3409)  [EEA28685.1](http://p450.riceblast.snu.ac.kr/class.php?a=dv_sequence&id=11997&spe_id=7910&ref_id=3409)  [EEA28678.1](http://p450.riceblast.snu.ac.kr/class.php?a=dv_sequence&id=11995&spe_id=7910&ref_id=3409) | CYP65Z2  CYP530A5  CYP551A2  CYP5042B1P | 4-hydroxybenzoate polyprenyl transferase (PMAA03470) and a C2H2 transcription factor | Ubiquinone biosynthesis |
| PMAA_031320  PMAA_031360 | [EEA28318.1](http://p450.riceblast.snu.ac.kr/class.php?a=dv_sequence&id=11985&spe_id=7910&ref_id=3409)  [EEA28322.1](http://p450.riceblast.snu.ac.kr/class.php?a=dv_sequence&id=11986&spe_id=7910&ref_id=3409) | CYP5082A3  CYP5093A1 | NRPS and gliotoxin biosynthesis genes | Secondary metabolite production – Gliotoxin^S^ |
| PMAA_063570  PMAA_063650 | [EEA25395.1](http://p450.riceblast.snu.ac.kr/class.php?a=dv_sequence&id=12006&spe_id=7910&ref_id=3409)  [EEA25248.1](http://p450.riceblast.snu.ac.kr/class.php?a=dv_sequence&id=12005&spe_id=7910&ref_id=3409) | CYP584E8  CYP62A1 | PKS and C6 transcription factor | Secondary metabolite production^S^ |
| PMAA_043610  PMAA_043600  PMAA_043670  PMAA_043550  PMAA_043630 | [EEA20527.1](http://p450.riceblast.snu.ac.kr/class.php?a=dv_sequence&id=12066&spe_id=7910&ref_id=3409)  [EEA20526.1](http://p450.riceblast.snu.ac.kr/class.php?a=dv_sequence&id=12065&spe_id=7910&ref_id=3409)  [EEA20533.1](http://p450.riceblast.snu.ac.kr/class.php?a=dv_sequence&id=12068&spe_id=7910&ref_id=3409)  [EEA20521.1](http://p450.riceblast.snu.ac.kr/class.php?a=dv_sequence&id=12064&spe_id=7910&ref_id=3409)  [EEA20529.1](http://p450.riceblast.snu.ac.kr/class.php?a=dv_sequence&id=12067&spe_id=7910&ref_id=3409) | CYP54C2  CYP570H1  CYP660A2  CYP503B4  CYP660B1 | bZIP transcription factor and toxin biosynthesis protein Tri7 (PMAA43560) | Secondary metabolite production |
| PMAA_050330  PMAA_050350  PMAA_050410 | [EEA21215.1](http://p450.riceblast.snu.ac.kr/class.php?a=dv_sequence&id=12055&spe_id=7910&ref_id=3409)  [EEA21217.1](http://p450.riceblast.snu.ac.kr/class.php?a=dv_sequence&id=12056&spe_id=7910&ref_id=3409)  [EEA21223.1](http://p450.riceblast.snu.ac.kr/class.php?a=dv_sequence&id=12057&spe_id=7910&ref_id=3409) | CYP584E8  CYP617A2  CYP585A1 | NRPS and a PKS | Secondary metabolite production^S^ |
| PMAA_088100  PMAA_088180  PMAA_088170 | [EEA24836.1](http://p450.riceblast.snu.ac.kr/class.php?a=dv_sequence&id=12026&spe_id=7910&ref_id=3409)  [EEA24844.1](http://p450.riceblast.snu.ac.kr/class.php?a=dv_sequence&id=12028&spe_id=7910&ref_id=3409)  EEA24843.1 | CYP68L1  CYP584E2  CYP58M2 | NPRS and a PKS | Secondary metabolite production^S^ |
| PMAA_031520  PMAA_031530 | [EEA28338.1](http://p450.riceblast.snu.ac.kr/class.php?a=dv_sequence&id=11987&spe_id=7910&ref_id=3409)  EEA28339.1 | CYP5282C1  CYP584 | PKS and a secondary metabolism cluster | Secondary metabolite production^S^ |
| PMAA_025880  PMAA_025930 | [EEA27738.1](http://p450.riceblast.snu.ac.kr/class.php?a=dv_sequence&id=11979&spe_id=7910&ref_id=3409)  [EEA27743.1](http://p450.riceblast.snu.ac.kr/class.php?a=dv_sequence&id=11980&spe_id=7910&ref_id=3409) | CYP5171A1  CYP682H1 | C6 transcription factor |  |
| PMAA_098630  PMAA_098650 | [EEA23273.1](http://p450.riceblast.snu.ac.kr/class.php?a=dv_sequence&id=12041&spe_id=7910&ref_id=3409)  [EEA23275.1](http://p450.riceblast.snu.ac.kr/class.php?a=dv_sequence&id=12042&spe_id=7910&ref_id=3409) | CYP504E2  CYP684A4 | C6 transcription factor |  |
| PMAA_085890  PMAA_085840 | [EEA24596.1](http://p450.riceblast.snu.ac.kr/class.php?a=dv_sequence&id=12023&spe_id=7910&ref_id=3409)  [EEA24591.1](http://p450.riceblast.snu.ac.kr/class.php?a=dv_sequence&id=12022&spe_id=7910&ref_id=3409) | CYP504E1  CYP5128A2 |  |  |
| PMAA_007450  PMAA_007490  PMAA_007440 | [EEA19978.1](http://p450.riceblast.snu.ac.kr/class.php?a=dv_sequence&id=12080&spe_id=7910&ref_id=3409)  [EEA19982.1](http://p450.riceblast.snu.ac.kr/class.php?a=dv_sequence&id=12081&spe_id=7910&ref_id=3409)  [EEA19977.1](http://p450.riceblast.snu.ac.kr/class.php?a=dv_sequence&id=12079&spe_id=7910&ref_id=3409) | CYP65AF1  CYP575A1  CYP617D6 |  |  |
| PMAA_097910  PMAA_097900  PMAA_097880 | [EEA23202.1](http://p450.riceblast.snu.ac.kr/class.php?a=dv_sequence&id=12039&spe_id=7910&ref_id=3409)  [EEA23201.1](http://p450.riceblast.snu.ac.kr/class.php?a=dv_sequence&id=12038&spe_id=7910&ref_id=3409)  [EEA23199.1](http://p450.riceblast.snu.ac.kr/class.php?a=dv_sequence&id=12037&spe_id=7910&ref_id=3409) | CYP5075A2  CYP5090A1  CYP606B2 |  |  |
| PMAA_032120  PMAA_032150 | [EEA28399.1](http://p450.riceblast.snu.ac.kr/class.php?a=dv_sequence&id=11990&spe_id=7910&ref_id=3409)  [EEA28402.1](http://p450.riceblast.snu.ac.kr/class.php?a=dv_sequence&id=11991&spe_id=7910&ref_id=3409) | CYP617D8  CYP5053C1 |  |  |
| PMAA_092970  PMAA_093020 | [EEA22677.1](http://p450.riceblast.snu.ac.kr/class.php?a=dv_sequence&id=12033&spe_id=7910&ref_id=3409)  [EEA22682.1](http://p450.riceblast.snu.ac.kr/class.php?a=dv_sequence&id=12034&spe_id=7910&ref_id=3409) | CYP531E1  CYP5104B2 |  |  |
| PMAA_070450  PMAA_070520 | [EEA25958.1](http://p450.riceblast.snu.ac.kr/class.php?a=dv_sequence&id=12011&spe_id=7910&ref_id=3409)  [EEA25965.1](http://p450.riceblast.snu.ac.kr/class.php?a=dv_sequence&id=12012&spe_id=7910&ref_id=3409) | CYP619B2  CYP5107B1 |  |  |
| PMAA_100700  PMAA_100660 | [EEA23482.1](http://p450.riceblast.snu.ac.kr/class.php?a=dv_sequence&id=12048&spe_id=7910&ref_id=3409)  [EEA23478.1](http://p450.riceblast.snu.ac.kr/class.php?a=dv_sequence&id=12047&spe_id=7910&ref_id=3409) | CYP59D3  CYP5266B1 |  |  |
| PMAA_033360  PMAA_033280 | [EEA28529.1](http://p450.riceblast.snu.ac.kr/class.php?a=dv_sequence&id=11993&spe_id=7910&ref_id=3409)  [EEA28521.1](http://p450.riceblast.snu.ac.kr/class.php?a=dv_sequence&id=11992&spe_id=7910&ref_id=3409) | CYP620B2  CYP614A2 |  |  |
| PMAA_009440  PMAA_009430  PMAA_009420 | [EEA18658.1](http://www.ncbi.nlm.nih.gov/protein/210064563)  [EEA18657.1](http://www.ncbi.nlm.nih.gov/protein/210064562)  [EEA18656.1](http://www.ncbi.nlm.nih.gov/protein/210064561) | CYP65B2  CYP65V6  CYP638A2 |  | Secondary metabolite production^S^ |
| PMAA_101770 | [EEA23592.1](http://p450.riceblast.snu.ac.kr/class.php?a=dv_sequence&id=12052&spe_id=7910&ref_id=3409) | CYP5041A2 | PKS and MFS transporter | Secondary metabolite production^S^ |
| PMAA_001070 | [EEA19307.1](http://p450.riceblast.snu.ac.kr/class.php?a=dv_sequence&id=12075&spe_id=7910&ref_id=3409) | CYP5195B1 | PKS and NRPS | Secondary metabolite production^S^ |
| PMAA_062960 | [EEA25175.1](http://p450.riceblast.snu.ac.kr/class.php?a=dv_sequence&id=12003&spe_id=7910&ref_id=3409) | CYP5077B1 | PKS and NRPS | Secondary metabolite production |
| PMAA_101680 | [EEA23583.1](http://p450.riceblast.snu.ac.kr/class.php?a=dv_sequence&id=12051&spe_id=7910&ref_id=3409) | CYP5116A1 | PKS | Secondary metabolite production |
| PMAA_020360 | [EEA27145.1](http://p450.riceblast.snu.ac.kr/class.php?a=dv_sequence&id=11975&spe_id=7910&ref_id=3409) | CYP539J1 | NRPS | Secondary metabolite production^S^ |
| PMAA_031760 | EEA28362.1 | CYP548A2 | PKS and MFS transporter | Secondary metabolite production |
| PMAA_038590 | EEA29077.1 | CYP620E3 | C6 transcription factor and metabolism genes | Quinic acid utilization |
| PMAA_065360 | [EEA25426.1](http://p450.riceblast.snu.ac.kr/class.php?a=dv_sequence&id=12007&spe_id=7910&ref_id=3409) | CYP5068C1 | Trichodiene synthase (PMAA_065350) | Secondary metabolite production - Terpenoids |
| PMAA_054540 | [EEA21658.1](http://p450.riceblast.snu.ac.kr/class.php?a=dv_sequence&id=12059&spe_id=7910&ref_id=3409) | CYP578C2 | Siderophore biosynthesis gene (PMAA_054480) | Secondary metabolite production - Siderophore biosynthesis |
| PMAA_071760 | EEA26095.1 | CYP5076A3 |  | Secondary metabolite production - Siderophore biosynthesis {Kelly, 2009 #445} |

* Putative CYP name as Besthit of Nelson’s was >40% identity

^S^ Located in predicted secondary metabolite clusters by SMURF (http://jcvi.org/smurf/index.php)
