# Supplementary material for: Talaromyces marneffei simA Encodes a Fungal Cytochrome P450 Essential for Survival in Macrophages
Source: mSphere. 2018 Mar 21;3(2):e00056-18. doi: 10.1128/mSphere.00056-18 (PMC5863032; doi:10.1128/mSphere.00056-18)
Supplement: FIG S1 [file sph002182498sf1.pdf]

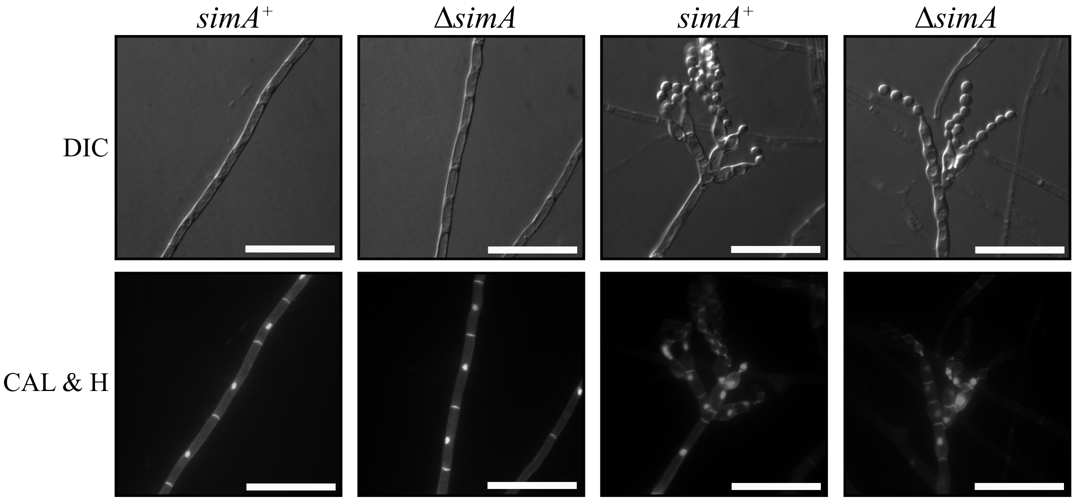

Supplementary Figure 1. The Δ*simA* mutant produces hyphae and conidiophores with wildtype morphology at 25ºC.

The wildtype (*simA*+) and Δ*simA* strains grown on ANM containing 1% or 0.1% glucose for 4 days at 25ºC. Images were captured using differential interference contrast (DIC) or with epifluorescence to observe calcofluor stained fungal cell walls (CAL) and Hoechst 33258 stained nuclei (Hoescht). Scale bars, 20µm.
