# Supplementary material for: Talaromyces marneffei simA Encodes a Fungal Cytochrome P450 Essential for Survival in Macrophages
Source: mSphere. 2018 Mar 21;3(2):e00056-18. doi: 10.1128/mSphere.00056-18 (PMC5863032; doi:10.1128/mSphere.00056-18)
Supplement: FIG S2 [file sph002182498sf2.pdf]

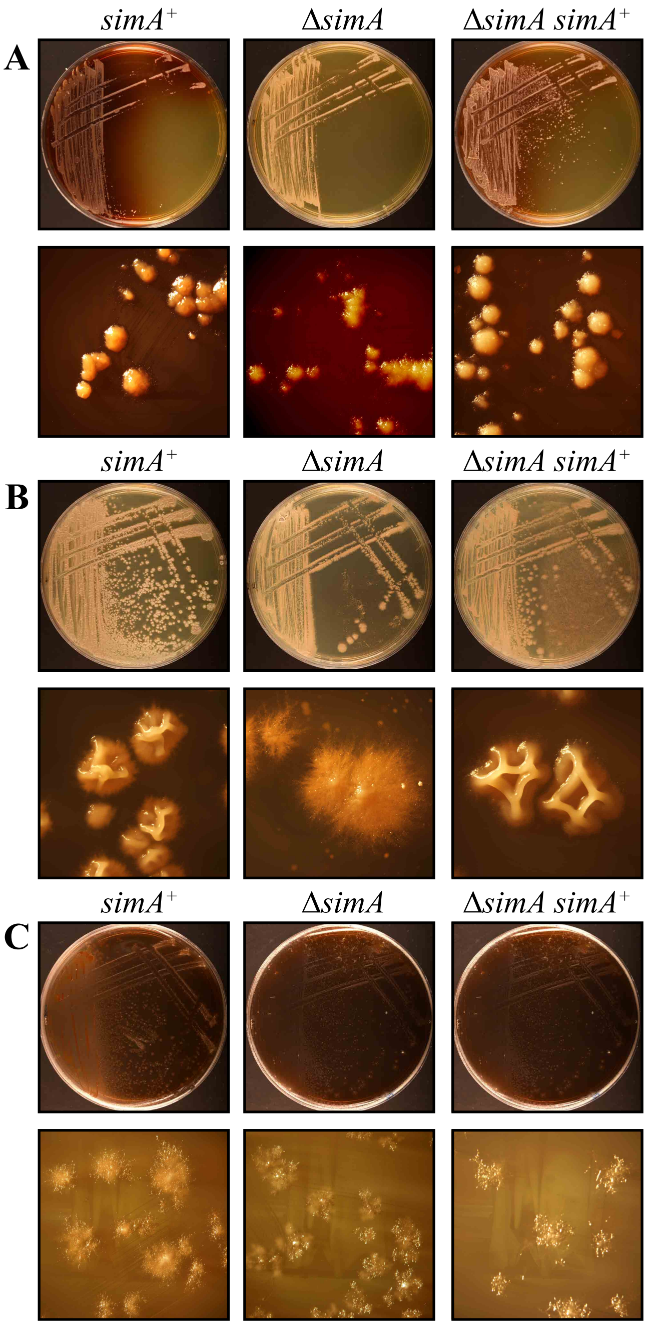

Supplementary Figure 2. The Δ*simA* mutant does not display a reduction in pyomelanin or DOPA melanization at 37ºC.
Growth of the wildtype, Δ*simA* and Δ*simA simA*+ strains at 37ºC on BHI medium after 5 days (A), tyrosine as the sole nitrogen source after 14 days (B) and on L-DOPA medium after 14 days (C).
