# Supplementary material for: Talaromyces marneffei simA Encodes a Fungal Cytochrome P450 Essential for Survival in Macrophages
Source: mSphere. 2018 Mar 21;3(2):e00056-18. doi: 10.1128/mSphere.00056-18 (PMC5863032; doi:10.1128/mSphere.00056-18)
Supplement: TABLE S4 [file sph002182498st4.doc]

**Supplementary Table 4. Polar metabolites showing a significant difference in levels between mid-log yeast cells of wildtype and the Δ*simA* mutant**

| **Metabolite** | **Score** | **BH Adj** | **Identification** |
| --- | --- | --- | --- |
| 116.75.6.414.91 | 0 | 0 | Alanine |
| 102.75.7.421.28 | 0 | 0 | Glycine |
| 130.75.9.438.25 | 0 | 0 | N-ethyl glycine |
| 241.133.22.440.38 | 0 | 0 | Monomethyl phosphate |
| 102.75.22.445.64 | 0 | 0 | beta-Alanine |
| 144.75.11.453.46 | 0 | 0 | Valine |
| 116.75.30.468.44 | 0 | 0 | Serine pk1 |
| 205.117.62.470.43 | 0 | 0 | Glycerol |
| 117.84.69.476.21 | 0 | 0 | 1,2,3-butanetriol |
| 158.75.6.479.79 | 0 | 0 | Isoleucine |
| 117.75.47.481.26 | 0 | 0 | Threonine |
| 218.100.14.520.58 | 0 | 0 | N/A |
| 189.133.80.521.83 | 0 | 0 | N/A |
| 218.128.23.528.35 | 0 | 0 | Homoserine |
| 128.100.55.534.17 | 0 | 0 | N/A |
| 174.128.34.534.54 | 0 | 0 | N/A |
| 217.103.86.541.32 | 0 | 0 | Threitol pk1 |
| 217.205.69.543.94 | 0 | 0 | Threitol pk2 |
| 218.217.91.545.58 | 0 | 0 | N/A |
| 75.70.5.548.46 | 0 | 0 | Nicotinamide |
| 176.84.91.553.08 | 0 | 0 | Methionine |
| 117.103.58.554.20 | 0 | 0 | N/A |
| 218.205.26.562.75 | 0 | 0 | N/A |
| 218.103.73.571.52 | 0 | 0 | N/A |
| 84.75.12.579.40 | 0 | 0 | Glutamate |
| 174.82.13.584.85 | 0 | 0 | 5-amino valeric acid |
| 218.192.78.586.31 | 0 | 0 | Phenylalanine pk2 |
| 217.103.73.605.07 | 0 | 0 | Ribitol |
| 157.142.42.635.86 | 0 | 0 | N/A |
| 199.75.53.639.02 | 0 | 0 | N/A |
| 319.205.78.659.56 | 0 | 0 | Mannitol |
| 154.104.49.662.26 | 0 | 0 | Histidine |
| 319.205.64.662.38 | 0 | 0 | Sorbitol |
| 218.100.9.666.76 | 0 | 0 | Tyrosine |
| 217.204.54.684.60 | 0 | 0 | N/A |
| 142.75.57.685.83 | 0 | 0 | N/A |
| 217.205.84.707.63 | 0 | 0 | N/A |
| 174.103.35.708.94 | 0 | 0 | N/A |
| 75.74.33.711.31 | 0 | 0 | N/A |
| 128.75.19.719.72 | 0 | 0 | N/A |
| 218.128.76.726.15 | 0 | 0 | N/A |
| 319.217.30.726.82 | 0 | 0 | N/A |
| 204.103.24.741.10 | 0 | 0 | N/A |
| 204.75.12.747.58 | 0 | 0 | N/A |
| 204.103.19.749.32 | 0 | 0 | N/A |
| 387.315.62.753.28 | 0 | 0 | Phospho-sugar(?) |
| 217.103.67.835.48 | 0 | 0 | N/A |
| 204.103.31.858.62 | 0 | 0 | Disaccharide(?) |
| 217.204.81.860.24 | 0 | 0 | Disaccharide(?) |
| 117.75.27.399.89 | 0.0001 | 0.000381159 | Lactic acid |
| 89.59.71.434.48 | 0.0001 | 0.000381159 | N/A |
| 174.100.20.471.24 | 0.0001 | 0.000381159 | Glycerol |
| 205.102.95.514.49 | 0.0001 | 0.000381159 | N/A |
| 160.75.67.523.41 | 0.0001 | 0.000381159 | Aspartate pk1 |
| 217.103.41.547.23 | 0.0001 | 0.000381159 | N/A |
| 84.75.63.557.07 | 0.0001 | 0.000381159 | N/A |
| 174.148.24.559.90 | 0.0001 | 0.000381159 | N/A |
| 120.75.38.567.00 | 0.0001 | 0.000381159 | Phenylalanine pk1 |
| 142.70.49.578.53 | 0.0001 | 0.000381159 | N/A |
| 260.128.37.605.90 | 0.0001 | 0.000381159 | N/A |
| 103.89.8.644.79 | 0.0001 | 0.000381159 | Fructose pk1 |
| 174.156.13.646.37 | 0.0001 | 0.000381159 | N/A |
| 154.103.33.663.70 | 0.0001 | 0.000381159 | N/A |
| 156.75.24.672.72 | 0.0001 | 0.000381159 | N/A |
| 156.154.4.695.65 | 0.0001 | 0.000381159 | N/A |
| 217.204.95.755.29 | 0.0001 | 0.000381159 | N/A |
| 84.75.16.774.96 | 0.0001 | 0.000381159 | N/A |
| 174.86.22.524.08 | 0.0002 | 0.000710811 | 3-aminoisobutyric acid |
| 218.205.72.574.10 | 0.0002 | 0.000710811 | N/A |
| 234.128.72.593.70 | 0.0002 | 0.000710811 | N/A |
| 305.217.92.701.51 | 0.0002 | 0.000710811 | myo-Inositol |
| 202.200.2.732.28 | 0.0002 | 0.000710811 | Tryptophan |
| 75.59.59.443.05 | 0.0003 | 0.001011538 | N/A |
| 218.189.70.489.42 | 0.0003 | 0.001011538 | Glyceric acid |
| 217.103.98.592.78 | 0.0003 | 0.001011538 | N/A |
| 361.217.37.817.14 | 0.0003 | 0.001011538 | Maltose pk1 |
| 213.107.33.721.41 | 0.0004 | 0.001331646 | Glutathione reduced |
| 185.59.75.564.31 | 0.0007 | 0.00230125 | N/A |
| 174.75.77.397.21 | 0.0008 | 0.00253494 | Pyruvate |
| 117.103.83.574.83 | 0.0008 | 0.00253494 | N/A |
| 217.131.79.862.48 | 0.0008 | 0.00253494 | Uridine monophosphate |
| 188.172.81.446.57 | 0.001 | 0.002988636 | N/A |
| 218.205.33.530.06 | 0.001 | 0.002988636 | N/A |
| 156.74.17.704.12 | 0.001 | 0.002988636 | N/A |
| 243.74.87.817.09 | 0.001 | 0.002988636 | N/A |
| 204.191.23.866.60 | 0.001 | 0.002988636 | N/A |
| 243.169.38.683.66 | 0.0011 | 0.003250562 | Cytidine monophosphate |
| 158.102.6.472.12 | 0.0012 | 0.003506667 | Leucine |
| 217.103.97.835.00 | 0.0013 | 0.003757143 | N/A |
| 169.74.15.897.89 | 0.0017 | 0.004859783 | Adenosine monophosphate |
| 117.83.64.555.70 | 0.002 | 0.005655914 | N/A |
| 70.66.4.536.41 | 0.0021 | 0.005875532 | N/A |
| 116.75.18.411.03 | 0.0023 | 0.006367368 | N/A |
| 361.217.80.818.04 | 0.0024 | 0.006575 | N/A |
| 142.100.7.633.95 | 0.0025 | 0.006778351 | Ornithine |
| 218.204.42.518.86 | 0.0028 | 0.007514286 | N/A |
| 245.217.31.641.24 | 0.003 | 0.007969697 | N/A |
| 103.74.20.515.91 | 0.0032 | 0.008416 | N/A |
| 204.100.17.499.83 | 0.0038 | 0.00989505 | Serine pk2 |
| 155.83.92.557.59 | 0.004 | 0.010313725 | N/A |
| 142.75.4.483.67 | 0.0044 | 0.011234951 | Proline |
| 280.156.84.655.85 | 0.0048 | 0.012138462 | N/A |
| 217.103.53.597.48 | 0.0049 | 0.012273333 | N/A |
| 154.132.16.658.69 | 0.005 | 0.01240566 | N/A |
| 361.217.80.807.29 | 0.0051 | 0.012535514 | Sucrose |
| 103.75.51.649.54 | 0.0059 | 0.014367593 | N/A |
| 217.74.15.627.48 | 0.0069 | 0.016648624 | N/A |
| 116.75.32.594.87 | 0.007 | 0.016736364 | Asparagine |
| 218.146.85.501.65 | 0.0075 | 0.01777027 | N/A |
| 102.75.33.451.52 | 0.0081 | 0.019020536 | N/A |
| 217.204.74.839.35 | 0.0084 | 0.019550442 | N/A |
| 218.117.72.532.46 | 0.0086 | 0.019840351 | N/A |
| 142.70.68.631.33 | 0.0093 | 0.021268696 | N/A |
| 241.99.78.495.72 | 0.0096 | 0.021765517 | Uracil |
| 217.75.21.629.23 | 0.0113 | 0.025400855 | 3-phosphoglyceric acid |
| 75.74.54.564.87 | 0.0115 | 0.025631356 | N/A |
| 218.217.63.567.68 | 0.0122 | 0.026963025 | N/A |
| 218.117.68.508.31 | 0.0142 | 0.031121667 | Threonine pk2 |
| 129.103.53.705.39 | 0.0158 | 0.034342149 | N/A |
| 182.153.20.655.25 | 0.0167 | 0.0359784 | N/A |
| 75.74.39.388.32 | 0.0169 | 0.0359784 | N/A |
| 292.217.94.621.30 | 0.017 | 0.0359784 | N/A |
| 217.204.66.633.39 | 0.0171 | 0.0359784 | N/A |
| 179.75.20.656.65 | 0.0184 | 0.038406349 | Tyrosine |
| 185.75.83.570.29 | 0.0193 | 0.039967717 | N/A |
| 86.75.34.435.66 | 0.0196 | 0.040271875 | N/A |
| 75.74.49.449.80 | 0.0207 | 0.042202326 | N/A |
| 103.75.56.640.41 | 0.0209 | 0.042282308 | N/A |
| 218.217.63.565.90 | 0.0211 | 0.042361069 | N/A |
| 103.89.10.647.05 | 0.0213 | 0.042438636 | Fructose pk2 |
