# Supplementary material for: Talaromyces marneffei simA Encodes a Fungal Cytochrome P450 Essential for Survival in Macrophages
Source: mSphere. 2018 Mar 21;3(2):e00056-18. doi: 10.1128/mSphere.00056-18 (PMC5863032; doi:10.1128/mSphere.00056-18)
Supplement: TABLE S5 [file sph002182498st5.doc]

**Supplementary Table 5. Sterols showing a significant difference in levels between mid-log yeast cells of wildtype and the Δ*simA* mutant**

| **Metabolite** | **Score** | **BH Adj** | **Identification** |
| --- | --- | --- | --- |
| 217.129.41.339.03 | 0 | 0 | N/A |
| 129.75.30.343.99 | 0 | 0 | N/A |
| 217.117.59.345.43 | 0 | 0 | N/A |
| 149.74.38.345.84 | 0 | 0 | N/A |
| 289.156.63.362.95 | 0 | 0 | N/A |
| 241.129.99.433.03 | 0 | 0 | N/A |
| 75.69.59.948.80 | 0 | 0 | Ergosta-7,22-diene |
| 69.67.25.1018.74 | 0 | 0 | 24-methylene lanost-8-en-3-yl |
| 217.117.26.355.13 | 0.0002 | 0.003911111 | Palmitoleic acid |
| 71.69.17.341.11 | 0.0003 | 0.0044 | N/A |
| 218.156.11.372.86 | 0.0003 | 0.0044 | N/A |
| 217.191.47.520.33 | 0.0003 | 0.0044 | N/A |
| 221.217.45.339.14 | 0.0006 | 0.0077 | N/A |
| 293.220.26.349.50 | 0.0007 | 0.0077 | N/A |
| 289.199.16.551.11 | 0.0007 | 0.0077 | N/A |
| 299.211.69.822.40 | 0.0007 | 0.0077 | N/A |
| 327.89.29.383.96 | 0.0008 | 0.008282353 | Octadecan-1-ol |
| 217.103.60.544.68 | 0.0009 | 0.0088 | 3a-mannobiose (very putative) |
| 149.71.21.322.95 | 0.0015 | 0.013894737 | N/A |
| 71.69.32.438.12 | 0.0016 | 0.01408 | N/A |
| 71.69.26.351.48 | 0.0023 | 0.01927619 | N/A |
| 131.95.6.785.90 | 0.0027 | 0.0216 | N/A |
| 154.71.11.331.64 | 0.0029 | 0.022 | N/A |
| 222.87.16.429.25 | 0.003 | 0.022 | N/A |
| 149.71.17.489.53 | 0.0039 | 0.027377778 | N/A |
| 75.69.48.471.66 | 0.0041 | 0.027377778 | N/A |
| 307.149.68.317.97 | 0.0042 | 0.027377778 | N/A |
| 211.84.95.376.61 | 0.0046 | 0.028914286 | N/A |
| 217.103.38.533.97 | 0.0056 | 0.03344 | N/A |
| 376.251.37.971.43 | 0.0057 | 0.03344 | N/A |
| 129.75.23.469.46 | 0.0071 | 0.040309677 | N/A |
| 361.191.32.516.14 | 0.0074 | 0.040533333 | N/A |
| 221.207.46.1037.83 | 0.0076 | 0.040533333 | N/A |
| 71.69.33.704.93 | 0.0079 | 0.040894118 | N/A |
| 69.67.35.903.24 | 0.0086 | 0.043245714 | 5-dihydroergosterol |
| 69.67.20.930.02 | 0.0095 | 0.046444444 | N/A |
